# Redox‐Activated Probes Enable High‐Contrast Live Imaging of Native Postsynaptic Scaffolds

**DOI:** 10.1002/anie.202519933

**Published:** 2026-02-02

**Authors:** Christiane Huhn, Clémence Mille, Sheng‐Yang Ho, Felix Lützenkirchen, Vladimir Khayenko, Melanie Hein, Christian Werner, Matthias Kneussel, Johannes W. Hell, Christian G. Specht, Hans M. Maric

**Affiliations:** ^1^ Rudolf Virchow Center for Integrative and Translational Bioimaging University of Würzburg Würzburg Germany; ^2^ Biocenter, Department of Biotechnology and Biophysics University of Würzburg, Am Hubland Würzburg Germany; ^3^ NeuroBicêtre, Inserm U1195 Université Paris‐Saclay Le Kremlin‐Bicêtre France; ^4^ Department of Pharmacology University of California Davis Davis California USA; ^5^ Institute of Molecular Neurogenetics Center For Molecular Neurobiology Hamburg (ZMNH) University Medical Center Hamburg‐Eppendorf Hamburg Germany

**Keywords:** fluorescent probe, gephyrin, live‐cell imaging, neuron, peptide, PSD, synapse

## Abstract

Direct visualization of postsynaptic scaffolds in living neurons is essential for dissecting synaptic dynamics and plasticity. Existing methods for live synapse visualization have major constraints, relying on genetic engineering or multistep application of live‐cell incompatible antibodies or nanobodies. Available affinity probes and delivery strategies lack the required contrast due to incomplete or excess delivery. Here, we introduce Sylives, a set of compact, synthetic fluorescent peptides that enable high‐contrast live imaging of inhibitory (gephyrin) and excitatory (PSD‐95) postsynaptic scaffolds in native neurons. Critically, by pre‐purification of the redox‐cleavable CPP‐probe conjugate we overcome side‐product formation of in‐situ coupling strategies, achieving reliable cytosolic delivery and restored scaffold binding after intracellular reduction. The Sylive design addresses the need for nanomolar probe levels versus micromolar CPP for clean labelling and efficient delivery by decoupling targeting and uptake. Through quantitative evaluation of uptake and off‐target binding, we defined a transferrable parameter space for effective intracellular delivery. Near traceless Sylive uptake and target specificity are validated by direct comparison to transiently expressed proteins and immunolabeling in fixed neurons. The reduction‐sensitive Sylive conjugates enable high‐contrast, specificity‐restored labelling of endogenous postsynaptic sites without genetic modification and offer a modular platform for targeting alternative intracellular proteins in living primary neurons.

## Graphical abstract

Sylives are modified fluorescent peptides for high‐contrast live imaging of inhibitory (gephyrin) and excitatory (PSD‐95) postsynaptic scaffolds in native neurons. Clean labeling requires low nanomolar probe levels, whereas uptake needs micromolar cell‐penetrating peptide (CPP). Redox‐triggered CPP masking and release reconciles both, enabling efficient delivery with low‐background staining.

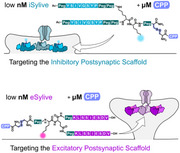

## 1 Introduction

Synapses relay electric and chemical signals between neurons, and their dysfunction is associated with neurological and psychiatric disorders [1, 2, 3, 4, 5]. The postsynaptic density (PSD) consists of highly concentrated scaffolding proteins, receptors, and signalling molecules, playing a crucial role in synaptic transmission and plasticity. Intracellular scaffolding proteins connect and cluster membrane receptors with intracellular effectors at the PSD, visualizing the spatial arrangement of scaffolding proteins is therefore crucial to understanding the regulation and plasticity of synaptic function [6]. The distribution and regulation of gephyrin, a key scaffolding protein in inhibitory post‐synapses, and PSD‐95 in excitatory post‐synapses has traditionally been studied using genetically encoded fluorescent tags and antibodies [7, 8, 9]. However, these methods are invasive and have significant practical limitations, particularly for live‐cell imaging. Fluorescent proteins suffer from less favourable photophysical properties, such as lower brightness, slower maturation and reduced photostability than synthetic fluorophores, which is essential for live‐cell imaging. Additionally, genetically encoded markers require transfection or viral transduction, which can alter cellular physiology, present issues with expression levels, and are not readily applicable to all experimental systems or species. Antibody‐based probes are generally incompatible with live‐cell imaging due to their size and poor membrane permeability. Existing small‐molecule methods have effectively labeled presynaptic sites via synaptic vesicle protein 2A [10, 11, 12] and postsynaptic sites using tools such as iSylite [13] and eSylite [14], whose application is limited to fixed samples. Small molecules that target neurotransmitter receptors [15, 16, 17], generally address extrasynaptic domains and are therefore largely restricted to surface receptors that rapidly exchange between synaptic and extrasynaptic sites and undergo activity‐related turnover, resulting in incomplete scaffold correlation. The activity of living synapses can be monitored using neurotransmitter‐dependent fluorescent dyes such as Fluo‐4, but these tools do not resolve individual or specific synapses [18, 19]. Thus, tools for high‐contrast imaging of functional synapses in living neurons are lacking, as available probes often only yield a transient snapshot of fluctuating neuronal surface proteins while the efficient delivery of fluorescent probes inside neurons remains challenging.

Viral transduction or knock‐in approaches are the gold standard for genetically encoded tags in neurons [3, 20]. Classical chemical transfection methods using cationic reagents show poor efficiency and high toxicity in neurons. Physical methods such as microinjection or optoporation [21] permit direct delivery but are low‐throughput and technically demanding. Streptolysin O (SLO) enables efficient cytosolic delivery of dyes and proteins in mammalian cells [22], but has not been successfully applied to neurons. In the field of neuroscience, acetoxymethyl esters are a popular tool to render biomolecules membrane permeable [23], but their application is restricted to the use of small molecules such as fluorophores and thus not directed against specific targets.

Intracellular delivery strategies using membrane‐interacting peptides and proteins have been recently refined and are increasingly applied successfully for the cytosolic delivery of macromolecular cargo [24, 25]. A specifically promising modular approach was introduced by Hackenberger and colleagues, combining polyarginine‐mediated uptake with cysteine‐based intracellular release of labeled proteins. These surface reactive peptides can interact with the cell membrane to enhance cellular uptake of therapeutic or functional biomolecules [26]. Surface reactive peptides often contain sequences that can bind to cell surface receptors or lipids, allowing them to anchor to specific cell membranes [27]. They typically exploit natural cellular entry pathways, such as endocytosis, or directly penetrate the lipid bilayer due to their amphipathic nature, which includes both hydrophilic and hydrophobic regions. Surface reactive peptides are currently considered among the most promising delivery systems for live cell imaging. First, they display high efficiency of intracellular delivery compared to traditional methods. Secondly, they cause limited toxicity by making use of natural cellular mechanisms and thereby minimizing membrane perturbation. Thirdly, surface reactive peptides are versatile and can be used to deliver a wide range of molecules, including small peptides, large proteins, nucleic acids, and even nanoparticles into cells.

**FIGURE 1:**
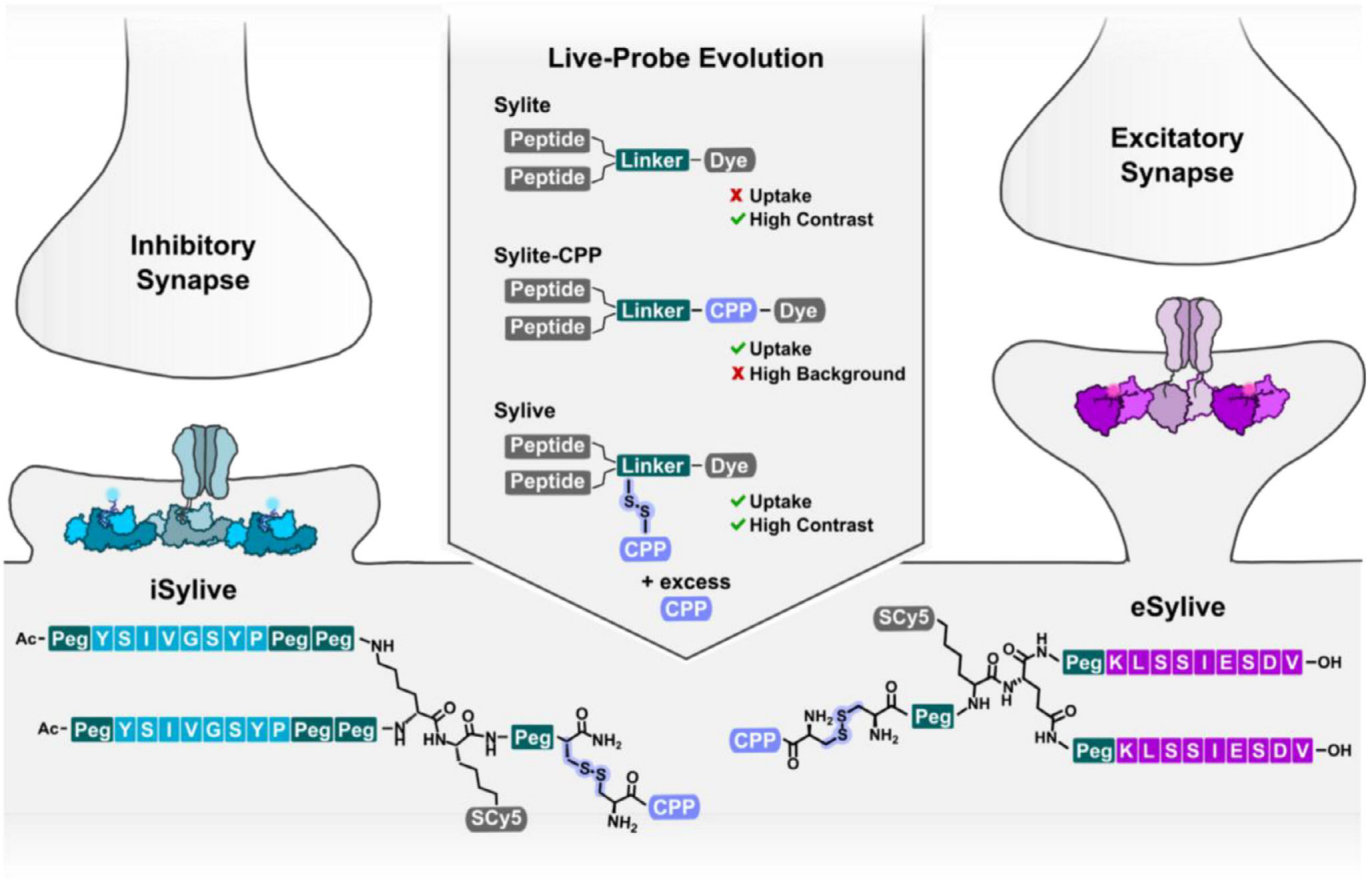
Sylives visualize inhibitory and excitatory postsynaptic sites in living neurons. Sylites are selective peptide probes for visualization of gephyrin, the scaffold of inhibitory synapses, and PSD‐95 in the excitatory synapse in fixed and permeabilized neurons [13, 14]. Live‐cell labelling using poly‐arginine derivatives of Sylites requires high concentrations and is ineffective. Sylives exploit surface reactive peptides to facilitate effective intracellular delivery and reversible binding, ensuring high‐contrast visualization.

Building on this concept, we overcome current barriers in live‐cell synapse visualization by developing an adapted method for compact, synthetic probes, enabling efficient and minimally invasive labeling of endogenous postsynaptic densities. Based on the selective scaffold binders iSylite and eSylite [14] that, while applied at low nanomolar concentration, were shown to induce physiological effects only at high micromolar concentrations [28, 29] we here developed Sylives, live synapse imaging probes targeting gephyrin at the inhibitory synapse (iSylive) and PSD‐95 at the excitatory synapse (eSylive) (Figure 1). Unlike neurotransmitter receptors, which rapidly exchange between synaptic and extrasynaptic sites, scaffolding proteins persist for hours, form a stable structural core, and occur in higher copy numbers, making them easier to target without altering receptor function or inducing endocytosis. Thus, Sylives target the enduring framework of the synapse, providing a consistent landmark for imaging.

Critically, we establish the first high‐contrast live visualization of both inhibitory and excitatory postsynaptic scaffolds via their endogenous proteins, enabled by pre‐purified redox‐cleavable cell penetrating peptide (CPP) conjugates that promote efficient neuronal uptake and traceless cytosolic release. By identifying and isolating the primarily active species and optimizing uptake while minimizing off‐target effects and endosomal trapping, we defined a transferable parameter window for direct high‐contrast live imaging of native postsynaptic scaffolds in primary cells (Figure 1).

## 2 Results and Discussion

### 2.1 Probe Design

**FIGURE 2:**
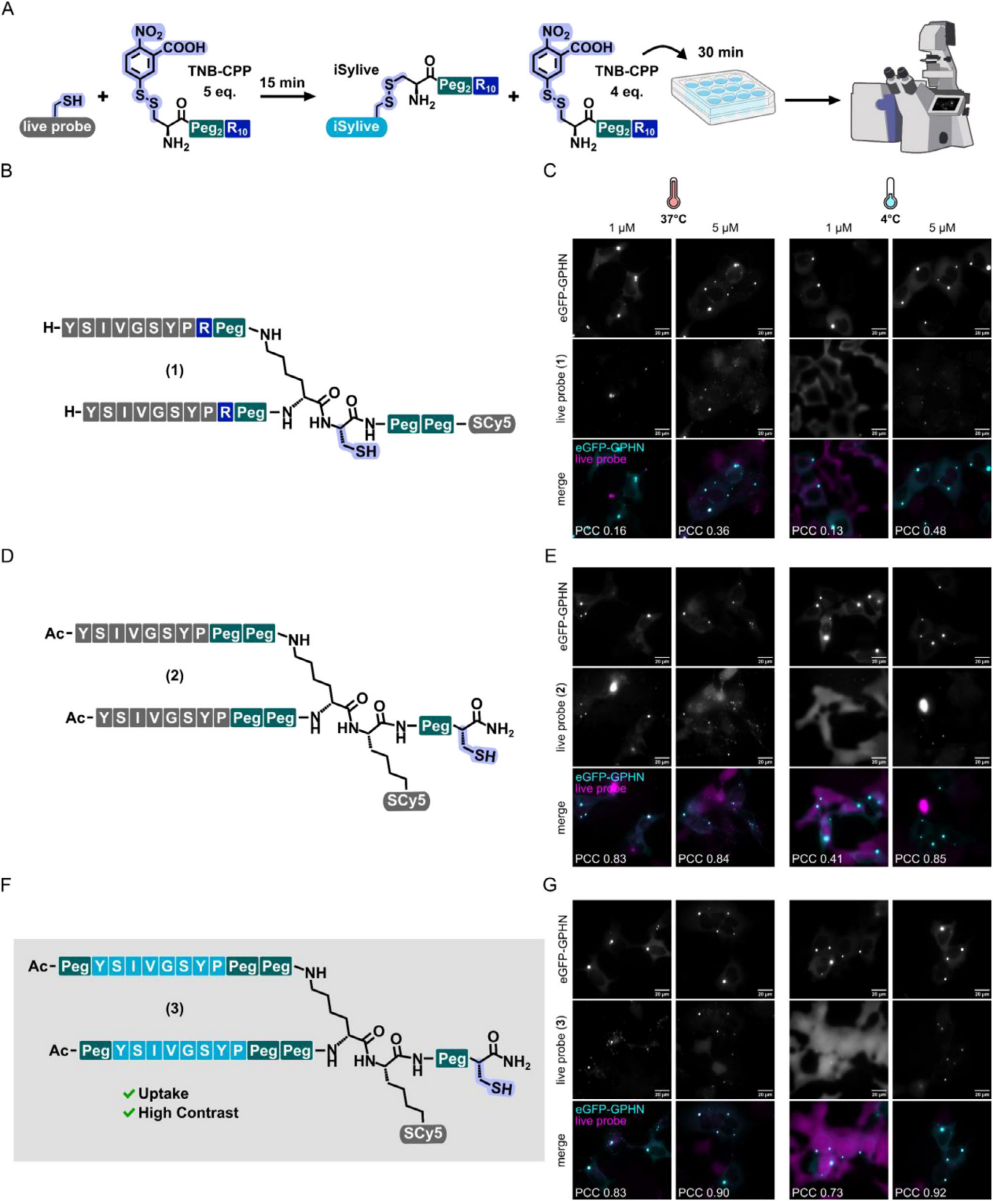
Definition of the structural requirements for effective cellular uptake. (A) Protocol for in situ conjugation of an excess of the TNB‐conjugated thiol‐reactive CPP with free thiol of the live probes. Preincubation is carried out for 15 min. The mixture is then applied to the living cells for 30 min prior to imaging. (B,D,F) Scheme of (B) live probe (**1**), (D) live probe (**2**) and (F) live probe (**3**) structure highlighting the changes made to the probe design based on iSylite. (C,E,G) Representative images of (C) live probe (**1**), (E) live probe (**2**) and (G) live probe (**3**) staining of HEK293 cells stably expressing eGFP‐gephyrin at 4°C or 37°C following 15 min co‐incubation with different concentrations of the live probe and 5 eq. of the TNB‐CPP, respectively. Note that the images at low temperature (4°C) and with low probe concentration (1 µM) are displayed with greater brightness to enhance the visibility of the non‐specific background labelling. PCC values are dominated by the large, bright clusters of the target protein.

As starting point for the development of a neuron‐specific peptide probe for live‐cell imaging, we chose a cytosolic delivery strategy that consists of in situ conjugation of the cargo molecule to an excess of thiol‐reactive CPP, in our case polyarginine, via a disulfide bond (Figure 2A). The use of an excess of the 5‐thio‐bis‐(2‐nitrobenzoic acid) (TNB) conjugated electrophilic thiol‐reactive CPP additive (TNB‐CPP, Figure S1A) allows covalent anchoring of the TNB‐CPP to the cell surface and thus a highly effective co‐delivery of the cargo at low micromolar concentrations by creating nucleation zones on the cell surface [26, 30, 31]. For a first probe design, the previously developed iSylite probe that is composed of a dimeric gephyrin‐targeting sequence and a fluorescent dye via cysteine maleimide chemistry [13] was evolved towards a live probe. Here, an additional PEG spacer and azide for fluorophore conjugation were added, while the cysteine retained a free thiol for in situ conjugation to a thiol‐reactive TNB‐CPP (Figures 2B and S1B). Labelling efficiency of this first live probe (**1**) was evaluated in living HEK293 cells that stably express eGFP‐tagged gephyrin (Figure 2C). In this assay, co‐localization of the peptide probe with large intracellular clusters of eGFP‐gephyrin is taken as a measure of internalization and specific binding to the target protein, which is dominated by the large, bright eGFP‐gephyrin clusters. The probe was pre‐incubated with 5 molar equivalents of TNB‐CPP for 15 min, and the mixture was then applied to the cells for 30 min at 37°C or 4°C. Results with the first live probe (**1**) were not convincing. Incubation at higher temperatures led to increased endosomal uptake, which is seen as a highly localized intracellular labeling that does not match the eGFP‐gephyrin puncta. In contrast, incubation at lower temperatures did not allow sufficient uptake of live probe (**1**) for high‐contrast imaging (Figures 2C and S2A). In this condition, only a very faint and non‐specific background staining of the entire cell was observed.

### 2.2 Structural Modifications for Improved Cellular Uptake

The initial probe architecture was revised to improve the access of the thiol for CPP conjugation. The cysteine was placed at the C‐terminus and linked with a PEG spacer to the dimeric probe that also contained two additional PEG spacers between the targeting sequences and the acetylation of the terminal amines that increase lipophilicity and biological stability [32] (Figures 2D and S1C, live probe (**2**)). At the same time, a variation of this probe was created (Figures 2F and SI S1D, live probe (**3**)), that possesses an additional PEGylation of the terminal amines to further reduce degradation by proteolytic enzymes [33]. Both probes were evaluated using the same pre‐incubation protocol as before at either 37°C or 4°C. For all tested probes and concentrations, the incubation of the living cells at 37°C led to increased endosomal uptake next to some specific signal that colocalized with eGFP‐gephyrin (Figures 2E,G, S2B,C, and S4). In contrast, incubation at 4°C gave high unspecific staining of the cells at lower concentrations of the live probe and TNB‐CPP, while increased concentration of the probe and TNB‐CPP produced a high degree of colocalization (Figure S4). To judge the specificity of the binding we measured the Pearson correlation coefficient (PCC) of live probe versus eGFP‐gephyrin. The PCC values were highest for live probe (**3**), qualifying it for further characterization.

### 2.3 Limitations of Oxidative Reversibly Binding Additives

**FIGURE 3:**
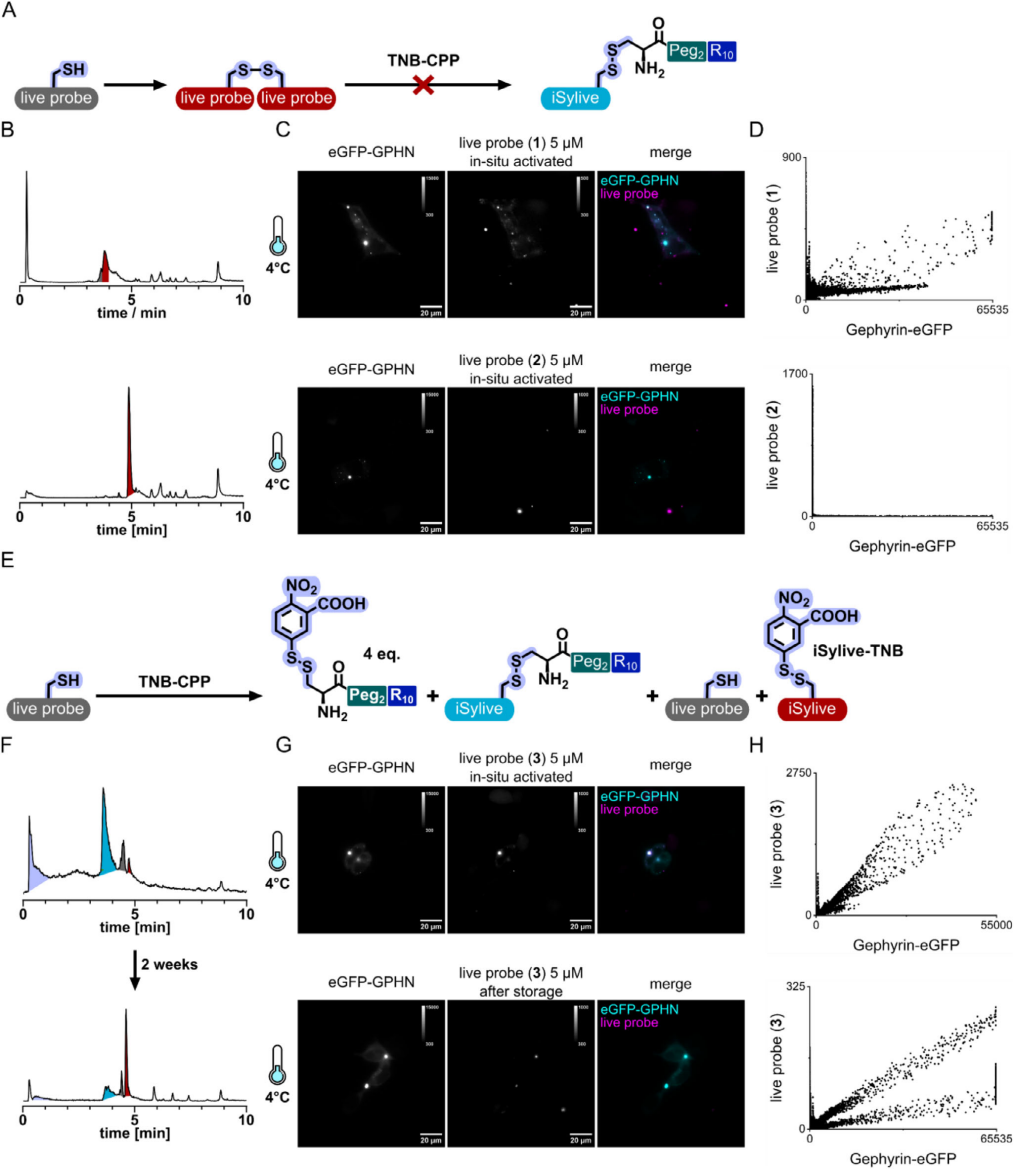
Factors limiting traceless uptake. (A) Reaction scheme of side‐product formation by live probe oxidation. (B) LC‐MS traces of live probes (**1**) and (**2**) after storage under oxidative conditions. Grey: Monomeric live probe, Red: dimeric live probe. (C) Representative images of oxidized live probe (**1**) and (**2**) staining of living HEK293 cells expressing eGFP‐gephyrin at 4°C following 15 min of incubation together with TNB‐CPP. (D) Cytofluorogram showing the intensity correlation of fluorescent‐tagged gephyrin versus affinity probe labelled gephyrin. (E) Reaction scheme of in situ conjugation of an excess of TNB‐CPP with the live probe highlighting all possible reaction products. (F) LC‐MS traces of reaction controls of in situ conjugation of live probe (**3**) with excess TNB‐CPP after 15 min and 2 weeks. Purple: TNB‐CPP, Cyan: iSylive‐CPP conjugate, Grey: unconjugated live probe (**3**), Red: TNB‐iSylive. (G) Sample images of live probe (**3**) staining of living HEK293 cells expressing eGFP‐gephyrin following 30 min of co‐incubation with TNB‐CPP and live probe (**3**) at 4°C. (H) Cytofluorogram of the respective live probe and eGFP‐gephyrin pixel intensities (arbitrary units, a.u.).

To optimize in situ conjugation towards the application of a small synthetic probe for high‐contrast imaging of living cells, we further analyzed the chemical reaction and formation of intermediates and side‐products during and after the co‐incubation of the TNB‐conjugated thiol‐reactive CPP and the iSylive probes. In this additive approach, thiol reactivity plays a crucial role for both creating nucleation zones on the cell surface by the TNB‐CPP additives as well as forming the actually active peptide‐CPP conjugates to enable efficient transduction. Thus, one major limitation of this approach is the oxidation sensitivity of the involved components. In the case of the iSylive probe, exposure to air or storage under oxidative conditions leads to dimerization of the probe via disulfide formation over time, yielding a probe that is unreactive towards the thiol‐reactive TNB‐CPP additive (Figure 3A). Application of oxidized probes on living HEK293 cells stably expressing eGFP‐gephyrin following the preincubation protocol showed only limited colocalization and aggregate formation depending on the oxidation progress that was monitored via LC‐MS (Figure 3B–D). Additionally, screening of the chemical reaction during in situ conjugation using LC‐MS revealed the formation of a side‐product over time (Figure 3E,F). Here, the CPP bound to the probe via disulfide bond is replaced by TNB (Figure S2A, iSylive‐TNB). Instead of enabling transduction of the probe, this modification renders it reactive towards the cell surface, which is accompanied by a loss of signal intensity and incomplete labelling of gephyrin in living HEK293 cells (Figure 3F,G).

### 2.4 The Isolated Active Probe Enables near Traceless Uptake

**FIGURE 4:**
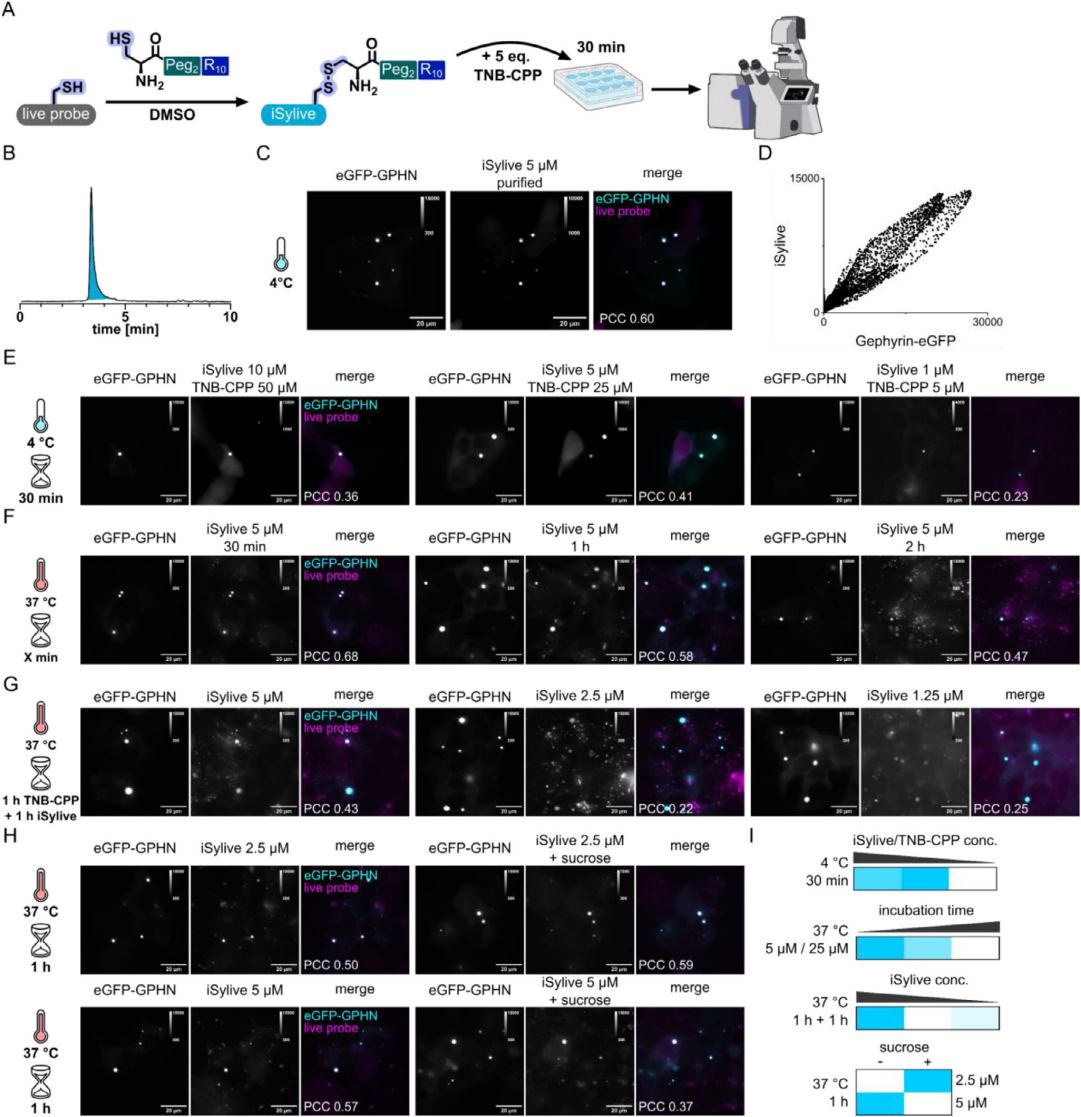
Improved uptake enabled by isolation of the activated iSylive probe and protocol optimization. (A) Reaction scheme of CPP‐iSylive conjugation and application on living cells. (B) LC‐MS traces of the isolated iSylive probe. (C) Representative images of iSylive staining of living HEK293 cells expressing eGFP‐gephyrin. The cells were directly stained with 5 µM iSylive and 25 µM TNB‐CPP for 30 min at 4°C. (D) Cytofluorogram of the iSylive and eGFP‐gephyrin signal intensities. (E) Representative images of iSylive staining of living HEK293 cells expressing eGFP‐gephyrin. The cells were directly stained with varying iSylive and TNB‐CPP concentrations for 30 min at 4°C. (F) Representative images of iSylive staining of living HEK293 cells expressing eGFP‐gephyrin. The cells were directly stained with 5 µM iSylive and 25 µM TNB‐CPP for varying incubation times at 37°C. (G) Representative images of iSylive staining of living HEK293 cells expressing eGFP‐gephyrin. The cells were incubated for 1 h with 25 µM TNB‐CPP before varying concentrations of iSylive were applied for another 1 h at 37°C. (H) Representative images of iSylive staining of living HEK293 cells expressing eGFP‐gephyrin. The cells were incubated with (right) or without (left) 450 mM sucrose in addition to varying concentrations of iSylive and 25 µM TNB‐CPP for 1 h at 37°C. (I) Heatmap of the PCC analysis of (E–H) normalized by max per experiment. Darker cyan indicates higher PCC with the max PCC represented in cyan and the min PCC in white. Arrangement is similar to the experimental data represented in (E–H).

To overcome the limitations of in situ conjugation of oxidative reversibly binding additives, namely oxidation sensitivity and side‐product formation, we isolated the actual active species composed of live probe bound to the CPP via a disulfide bond. Live probe (**3**) was coupled to the free cysteine of the CPP in DMSO, where thiol oxidation proceeds through an acid‐catalysed oxidation mechanism that generates disulfides without requiring molecular oxygen. The reaction yields the desired disulfide‐linked (**3**)‐CPP conjugate (Figure S2B, iSylive) as main product, which was subsequently purified to prevent side‐product formation (Figure 4A,B). The direct application of the resulting iSylive on living cells offers several advantages over the in situ conjugation with thiol‐reactive TNB‐CPP. No fluorescent side‐products are formed, such as the unconjugated live probe that is incapable of entering the cell on its own, or the TNB‐iSylive that can bind to the cell surface, causing extracellular background staining. The new probe still takes advantage of the reversible attachment of CPP via cysteine oxidation, releasing the iSylive probe in the reductive environment of the cytosol and enabling unhindered targeting. Direct application of iSylive together with an excess of the thiol‐reactive TNB‐CPP additive following the previous protocol allowed the visualization of gephyrin in living overexpressing HEK293 cells with superior contrast compared to the previous tested probes (Figure 4A,C,D).

### 2.5 Definition of Concentrations, Incubation Time, and Temperature for Improved Uptake

Next, we tested variations of the delivery protocol to optimize uptake of the new probe. Investigating several iSylive and TNB‐CPP concentrations with a 1:5 ratio at 4°C, the previously determined concentrations (5 µM live probe, 25 µM TNB‐CPP) yielded the highest PCC among the tested concentrations (Figure 4E). Applying the same conditions at 37°C led to specific staining with a similarly high intensity (Figure S5), however, this was accompanied by endosomal trapping of the probe after 30 min, and even more so after incubation for 1 h and 2 h at 37°C (Figure 4F). To reduce unspecific background staining, the minimal iSylive concentration was determined by preincubating the cells with 25 µM TNB‐CPP for 1 h before applying varying concentrations of the iSylive probe. Lowering the iSylive concentration led to a reduction of the specific staining and the prolonged incubation caused primarily endosomal accumulation of the probe (Figure 4G). To suppress endosomal uptake of the probe, sucrose was added in a subsequent experiment, which is known to block clathrin‐mediated endocytosis. The cells were incubated with 450 mM sucrose, 25 µM TNB‐CPP and iSylive at different concentrations for 1 h at 37°C. At 2.5 µM iSylive, the resulting images show a higher degree of colocalization and reduced endosomal signal, even though the specific signal is lower as well, indicating reduced uptake via both pathways. At 5 µM iSylive, the addition of sucrose causes increased background signal compared to the control lowering the PCC (Figure 4H). In total, these results indicate endosomal trap of the probe when incubated at 37°C over time, that can be reduced by either incubating the cells at 4°C or by addition of sucrose to suppress endosomal uptake, when longer acquisition times are needed for the respective application. PCC comparison of all tested conditions identified incubation of 5 µM iSylive together with 25 µM TNB‐CPP at both 4°C and 37°C for 30 min as the best conditions to achieve optimal uptake of the iSylive probe for high contrast microscopy, which we define as the practically useful imaging window (Figures 4I and S6).

### 2.6 Live Imaging of Inhibitory Postsynapses in Primary Hippocampal Neurons

**FIGURE 5:**
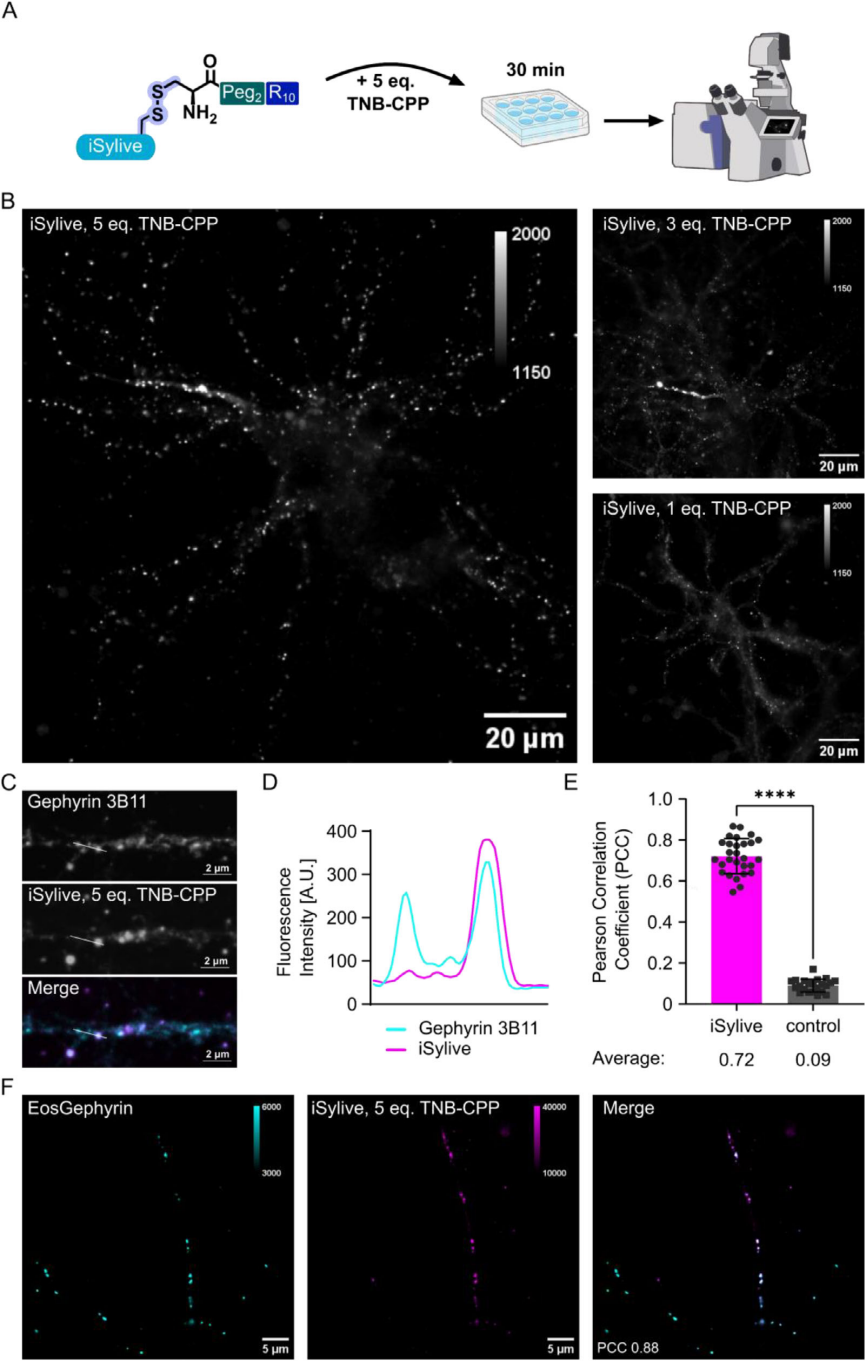
iSylive visualizes synapses in living mouse primary neurons. (A) Incubation protocol for live imaging of inhibitory synapses via the underlying endogenous scaffold using iSylive and TNB‐CPP additive. (B) Exemplary microscopy images of neurons live‐stained with varying concentrations of iSylive and TNB‐CPP. (C) iSylive labelling of endogenous gephyrin in primary hippocampal neurons to determine the ability of iSylive to visualize inhibitory postsynaptic structures in living neurons followed by fixation and gephyrin immunolabelling. (D) Line profile along a dendritic shaft region co‐labelled with iSylive (magenta) and anti‐gephyrin antibody (cyan). (E) Quantification of PCC in the presence of gephyrin antibody, TNB‐CPP and iSylive (magenta bar, left) compared to gephyrin antibody and TNB‐CPP only (control, grey bar right). (F) Live iSylive labelling of mEos4b‐gephyrin in lentivirus infected neurons, PCC 0.88 ± 0.03, mean ± SD, *n* = 7 cells.

We next explored the iSylive labelling efficiency of endogenous gephyrin in living primary neurons. We applied the previously defined protocol incubating the living primary neurons with iSylive (5 µM) and TNB‐CPP for 30 min at 4°C. To minimize the stress induced by membrane penetration through TNB‐CPP, we systematically varied the concentration of the TNB‐CPP additive. In line with the observations in eGFP‐gephyrin transfected HEK293 cells, the iSylive probe yielded the most robust labelling when co‐incubated with 5 equivalents of TNB‐CPP (Figure 5B), Notably, reducing the excess of TNP‐CPP led to a decline in cytosolic uptake and ultimately loss of effective synapse visualization, confirming that the TNB‐CPP additive is essential for effective delivery in primary neurons, highlighting the advantage of the reversible attachment of CPP separating probe concentration from uptake efficiency. Thus, the TNB‐CPP additive strategy not only improves delivery efficiency but also minimizes perturbation from the probe, while maintaining effective visualization of the postsynaptic scaffold in living neurons.

To confirm iSylive labelling of endogenous gephyrin, we incubated primary hippocampal neurons with 5 µM iSylive and 25 µM TNB‐CPP for 30 min at 4°C. The neurons were fixed, permeabilized, and co‐stained with the monoclonal gephyrin antibody 3B11 as reference. iSylive produced a punctate distribution of signals that specifically colocalized with endogenous gephyrin as visualized by the monoclonal gephyrin antibody (Figures 5C and S7A). Almost all antibody‐detected gephyrin puncta were recognized by iSylive, albeit with varying intensity. A line profile shows that some gephyrin peaks were only marked by weaker iSylive signals (Figure 5D). Additionally, the PCCs of endogenous gephyrin signals in the presence of TNB‐CPP and iSylive (Figure 5E, magenta bar) were compared with endogenous gephyrin signals in the presence of TNB‐CPP but lacking iSylive (control). A high degree of colocalization was exclusively observed with iSylive staining. Notably, the PCC value of 0.72 is only slightly below that obtained with the related iSylite probe in fixed neurons (0.86) [13], highlighting the effective uptake of the live probe.

To further validate iSylive visualization of gephyrin in living neurons, we transfected primary hippocampal neurons with mTom‐gephyrin and mRuby as a transfection control and applied the same iSylive protocol (5 µM iSylive, 25 µM TNB‐CPP, incubation for 30 min at 4°C) prior to live imaging. In both cases, a strong background in the form of probe aggregation was observed compared to untransfected neurons, indicating interference of the transfection agent with iSylive uptake (Figure S8B–D). To circumvent this perturbation, the neurons were instead transduced with a lentiviral construct driving the expression of mEos4b‐gephyrin and labelled with iSylive. To minimize the stress induced by low temperatures to the sensitive infected living neurons, we incubated 5 µM iSylive and 25 µM TNB‐CPP for 30 min at room temperature. The iSylive labelling showed a punctate distribution that colocalized with the mEos4b‐gephyrin signals, demonstrating efficient live‐cell targeting of the probe (Figures 5F and S7B). Again, colocalization remained high (mean PCC 0.88), although labelling intensities varied between puncta.

Together, these experiments establish that iSylive reliably labels endogenous gephyrin in both fixed and living neurons, as well as recombinant gephyrin in living neurons, providing an effective strategy for synapse visualization under physiological conditions.

### 2.7 Excitatory Postsynapse Visualization in Living Mouse Primary Neurons via PSD‐95

**FIGURE 6:**
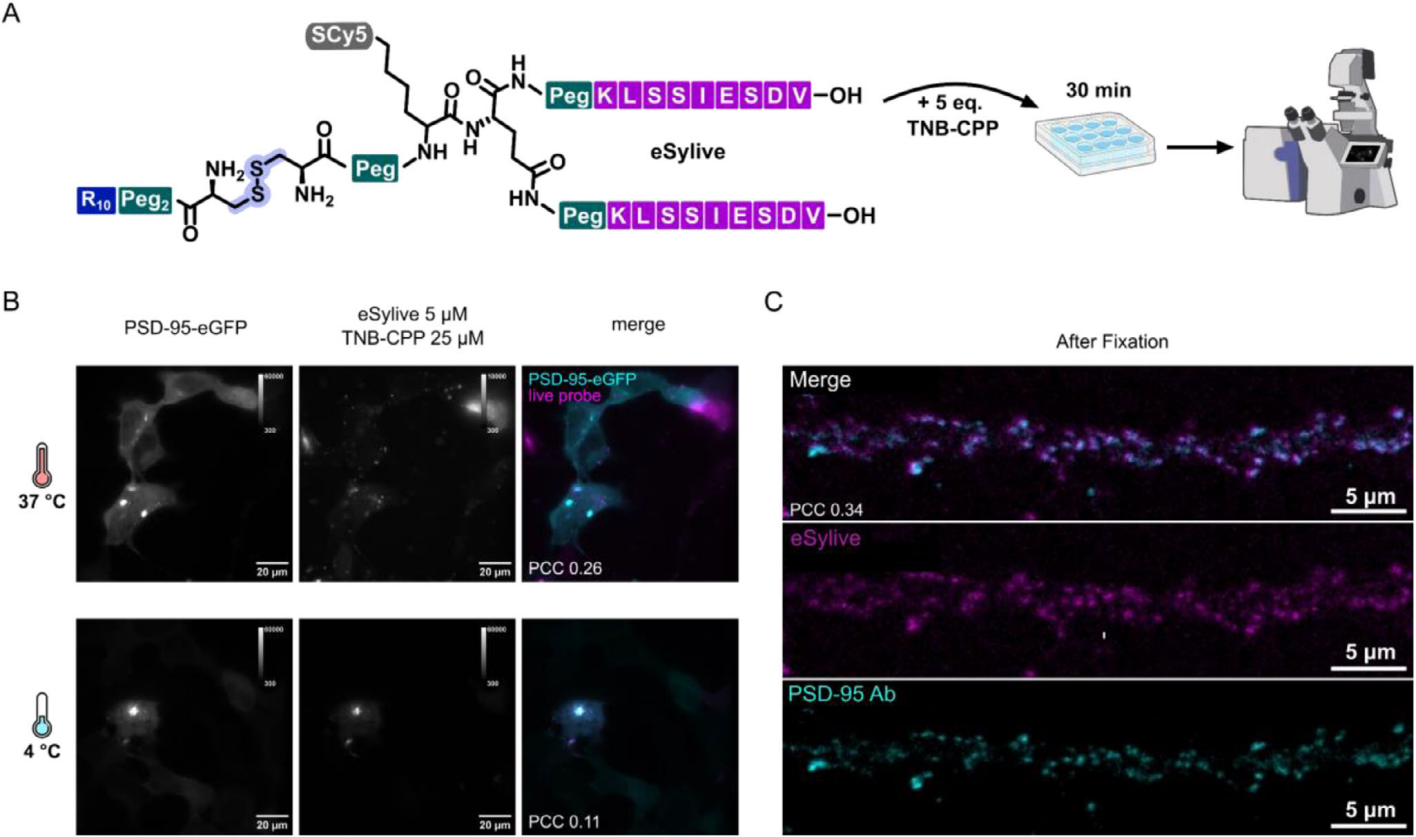
Visualization of excitatory synapses in living mouse primary neurons via PSD‐95 using eSylive. (A) eSylive probe design and incubation protocol for live visualization of excitatory synapses via the underlying endogenous scaffold using eSylive and TNB‐CPP additive. (B) Representative images of eSylive staining of living HEK293 cells expressing eGFP‐PSD‐95. The cells were directly stained with 5 µM eSylive and 25 µM TNB‐CPP for 30 min at either 37°C or 4°C. (C) eSylive labelling of endogenous neurons followed by fixation and PSD‐95 antibody co‐staining including several washing steps, before imaging by confocal microscopy.

To explore whether our labelling strategy can be applied to other synthetic probes for high‐contrast visualization of intracellular targets, we designed a live probe targeting the major scaffolding protein of the excitatory synapse, PSD‐95, based on a previously reported peptide label, eSylite [14]. This probe consists of a dimeric PSD‐95 binding sequence that provides the necessary free C‐terminus for target binding and is linked to the fluorophore via a cysteine at the N‐terminus. Following the structural requirements defined in this study, eSylite was evolved to eSylive by introducing a click handle for fluorophore conjugation at the N‐terminus, while retaining a free cysteine thiol for CPP coupling. To ensure CPP accessibility, the cysteine was connected via a PEG spacer. The final eSylive probe was obtained by oxidation to the CPP conjugate and subsequent purification (Figures 6A and S9).

Labelling efficiency was first evaluated in living HEK293 cells transfected with PSD‐95‐eGFP and incubated for 30 min at either 37°C or 4°C (Figures 6B and S10A,B) with 5 µM eSylive and five molar equivalents of TNB‐CPP. In both conditions, eSylive produced distinct staining of PSD‐95‐eGFP, accompanied by signal in the endosomal compartments at 37°C. However, increased background and probe aggregation was observed (Figure S11A) originating from the transfection in contrast to the eGFP‐gephyrin experiments in HEK293 cells, where the readout was based on stably expressing cells and thus yielded substantially higher PCC values. Additionally, probe performance of eSylite was shown to be sensitive towards small changes in the probe architecture, such as the choice of fluorophore and linkage [14]. Thus, the addition of strongly charged moieties such as the CPP might impact performance of the resulting live probe. To confirm endogenous PSD‐95 detection, primary hippocampal neurons were incubated with eSylive and TNB‐CPP for 30 min at 37°C eSylive labelling showed a punctate distribution consistent with the expected PSD‐95 pattern in hippocampal neurons, albeit accompanied by a higher background (Figure S11B). Because PSD‐95 differs from gephyrin in postsynaptic architecture, probe affinity and endogenous expression levels, the uptake and imaging parameters optimized for iSylive could not be directly transferred and required independent optimization. Systematic variation of probe and TNB‐CPP concentrations identified the incubation of 100 nM eSylive with 2 µM TNB‐CPP for 30 min at 37°C, followed by washout and recovery for 30 min as optimal for live imaging, balancing signal intensity and background (Figure S11B). Following fixation and antibody co‐staining under established conditions, eSylive colocalized extensively with PSD‐95 immunoreactivity, with nearly complete overlap under optimized conditions (Figures 6C, S10C, and S11C). These results demonstrate that eSylive provides effective access to PSD‐95 in living neurons, extending our probe strategy from inhibitory to excitatory synapses. Together with iSylive, this establishes a generalizable approach for live visualization of both inhibitory and excitatory synapses using compact synthetic probes.

## 3 Conclusion

In this study, we establish Sylives as compact, fully synthetic probes enabling high‐contrast real‐time visualization of native inhibitory and excitatory postsynaptic scaffolds in living neurons without genetic manipulation. By rationally targeting the core structural components of the postsynaptic density, Sylives provide minimally invasive access to synaptic organization and dynamics with molecular specificity and low perturbation. A central advancement of this work is the implementation of purified, redox‐cleavable CPP‐probe conjugates, which allow efficient membrane translocation and intracellular reductive release of the active probe without CPP remnants. Applying the CPP additive approach, the Sylive design allows separating probe concentration from uptake efficiency. This traceless mode of delivery overcomes major limitations of permanently CPP‐tagged constructs and previous in situ conjugation approaches, which can suffer from inconsistent stoichiometry, side‐product formation, reduced uptake, and diminished contrast. Importantly, quantitative analyses need to be performed under well‐defined, physiologically relevant conditions to ensure sufficient cytosolic redox potential and probe release. Pre‐purification of the disulfide‐linked conjugate proved critical, substantially improving labeling efficiency and imaging performance as validated by quantitative comparisons with tagged scaffolds in living overexpressing HEK293 cells, viral infected neurons and antibody‐based immunostaining after live labeling of endogenous neurons.

Transferring this strategy to PSD‐95, the scaffold protein of excitatory synapses, eSylive was successfully established, marking this approach as a general strategy for labeling intracellular structures for high‐contrast real‐time imaging. However, we note that eSylive currently exhibits a narrower optimal parameter window and lower signal intensity than iSylive, reflecting fundamental differences in excitatory versus inhibitory postsynaptic architecture, probe affinity, and endogenous scaffold expression levels. These factors necessitate target‐specific optimization but do not compromise the core design principle or applicability of the approach. Although endosomal signals can emerge following prolonged exposure, we show that short incubation, immediate washout, low probe concentrations, and optional inhibition of endocytosis effectively minimize endosomal trapping, yielding postsynaptic labeling suitable for dynamic studies. Compared to antibodies and their fragments, Sylives offer reduced molecular size, tunability, and compatibility with live‐cell imaging without genetic manipulation. Targeting functional binding sites, Sylives fit into a well‐established category of live‐cell probes including Lifeact for visualizing actin filaments [34], SiR‐Actin for F‐actin staining [35], and flumazenil‐based tracers for GABA receptors [22]. These probes are indispensable for real‐time visualization of dynamic cellular processes providing unique molecular specificity for endogenous structures. Their value is maximized when concentrations are chosen to balance imaging quality with minimal functional disruption. The Sylive probes build on receptor‐derived peptide motifs targeting postsynaptic scaffolds, which have been reported to induce physiological effects only when applied at high micromolar concentrations [28, 29] while enabling functional readouts of scaffold distribution and the availability of neurotransmitter receptor anchoring sites when applied at low concentrations. This allows researchers to investigate the precise organization of postsynapses and their changes, thus addressing a critical gap in our understanding of synaptic dynamics [29, 36].

The present work defines and validates key delivery parameters enabling quantitative live imaging of endogenous synaptic scaffolds in primary cultured neurons, which are the gold‐standard model for high‐resolution mechanistic studies of synaptic nano‐organization. Expansion to tissue slices or in vivo systems will require probe delivery strategies and imaging modalities capable of resolving single synapses, representing exciting future directions. Additionally, for long‐term imaging, possible interference from both the TNB‐CPP as well as the Sylives remain to be excluded.

Together, this study establishes Sylives as a robust, modular platform for high‐contrast, genetic‐free live‐cell imaging of postsynaptic structures. They extend the range of available tools for studying synaptic organization and dynamics in living neurons, bridging the gap between structural labeling and functional interrogation. Future studies will further explore the potential functional impact on synaptic physiology under varying conditions, and their adaptability to other intracellular protein complexes.

## Conflicts of Interest

The authors declare no conflicts of interest.

## Supporting information




**Supporting File 1**: anie71274‐sup‐0001‐SuppMat.docx.

## Data Availability

The data that support the findings of this study are available from the corresponding author upon reasonable request.
