# Supplementary material for: Redox‐Activated Probes Enable High‐Contrast Live Imaging of Native Postsynaptic Scaffolds
Source: Angew Chem Int Ed Engl. 2026 Feb 2;65(11):e19933. doi: 10.1002/anie.202519933 (PMC12970509; doi:10.1002/anie.202519933)

**Supplementary Material**

**Redox-Activated Probes Enable High-Contrast Live Imaging of Native Postsynaptic Scaffolds**

Christiane Huhn^1,2^, Clémence Mille^3^, Sheng-Yang Ho^4^, Felix Lützenkirchen^5^, Vladimir Khayenko^1,2^, Melanie Hein^1,2^, Christian Werner^2^, Matthias Kneussel^5^, Johannes W. Hell^4^, Christian G. Specht^3^, Hans M. Maric^1,2^*

^1^ Rudolf Virchow Center for Integrative and Translational Bioimaging, University of Würzburg, Josef-Schneider-Str. 2, 97080 Würzburg, Germany

^2^ University of Würzburg, Biocenter, Department of Biotechnology and Biophysics, Am Hubland, 97074 Würzburg, Germany

^3^ NeuroBicêtre, Inserm U1195, Université Paris-Saclay, 94276 Le Kremlin-Bicêtre, France

^4^ Department of Pharmacology, University of California Davis, Davis, CA, 95616, USA

^5^ Institute of Molecular Neurogenetics, Center for Molecular Neurobiology Hamburg (ZMNH), University Medical Center Hamburg-Eppendorf, Hamburg, Germany

*Correspondence to H.M. Maric (hans.maric@uni-wuerzburg.de)

**Table of Content**

[**Ethical Approval 2**](#_Toc216031505)

[**Materials and Methods 2**](#_Toc216031506)

[Solid Phase Peptide Synthesis 2](#_Toc216031507)

[Fluorophore conjugation 2](#_Toc216031508)

[CPP conjugation 2](#_Toc216031509)

[Purification and characterization of peptides 2](#_Toc216031510)

[HEK293 Cell Cultures and Transfection 2](#_Toc216031511)

[Live cell labelling of HEK293 cells stably expressing gephyrin-eGFP 3](#_Toc216031512)

[Wide Field Fluorescence Microscopy of HEK293 cells stably expressing gephyrin-eGFP 3](#_Toc216031513)

[Dissociated Hippocampal Neuronal Cultures 3](#_Toc216031514)

[Immunocytochemistry 4](#_Toc216031515)

[Confocal microscopy for fixed neuronal culture 4](#_Toc216031516)

[2D image processing and analysis 5](#_Toc216031517)

[**Supplementary Figures 6**](#_Toc216031518)

[Figure S1. iSylive and TNB-CPP chemical structures corresponding to Figure 2.](#_Toc216031520) 7

[Figure S2. iSylive chemical structures corresponding to Figure 3. 7](#_Toc216031522)

[Figure S3. eSylive chemical structures corresponding to Figure 6. 8](#_Toc216031525)

[Figure S4. Microscopic evaluation of iSylive probes (1) – (3) corresponding to Figure 2. 9](#_Toc216031528)

[Figure S5. Microscopic evaluation of iSylive at different incubation temperatures. 10](#_Toc216031530)

[Figure S6. iSylive application in transfected live neurons. 11](#_Toc216031532)

[Figure S7. eSylive application in untransfected live neurons. 12](#_Toc216031534)

[**Supplementary Tables 13**](#_Toc216031535)

[Supplementary Table 1. Mass spectrometric probe validation. 13](#_Toc216031536)

[**Supplementary References 14**](#_Toc216031537)

[**Appendix 1: Chromatographic and mass spectrometric probe validation 15**](#_Toc216031538)

**Ethical Approval**

Experiments were approved by the local veterinary authority. All procedures involving animals followed the guidelines for the Care and Use of Laboratory Animals from the US National Institutes of Health and were approved by the Institutional Animal Care and Use committees at the University of California, Davis. All procedures involving animals were carried out in accordance with institutional guidelines and approved by the local veterinary authorities (Regierung von Unterfranken, Würzburg, Germany; licence no. FBVVL 568/200-324/13; Inserm UMS44-Bicêtre; license G94043013).

**Materials and Methods**

Solid Phase Peptide Synthesis

The peptides were produced using standard solid phase peptide synthesis with Fmoc chemistry. 2-chlorotrityl resin (1.6 mmol/g) was swollen in dry DCM for 30 min, then, the desired amino acid (1 eq.), Boc-Gly-OH (1 eq.) and 4 eq. of dry DIEA in dry DCM were added to the resin slurry. After overnight reaction at RT with agitation, the resin was capped with MeOH and washed with DCM and DMF. Deprotection and conjugation cycles followed, where 20% piperidine solution in DMF was used to remove the Fmoc protecting group. After washes, the peptide chain was elongated by adding AA (4 eq.) with Oxyma (4 eq.) and DIC (4 eq.). Capping was done with DIEA (50 eq.) and acetic anhydride (50 eq.) in N-Methyl-2-pyrrolidone for 30 min. Coupling efficiency was monitored by measuring the light absorption of the dibenzofulvene–piperidine adduct in 20% pip solution after deprotection. The concentration in mM is determined from an absorption read at 290 nm in a quartz cuvette that has 1 cm long optical path. The peptides were cleaved from the resin using a cocktail of 90% TFA, 5% H2O, 5% Triisopropylsilane, for 2 to 4 hours at RT. Then, the peptides were precipitated in ice-cold ether and afterwards purified with reverse-phase HPLC and analyzed by LC-MS as described below.

Fluorophore conjugation

The peptides were conjugated to the fluorophore via copper-catalyzed azide-alkyne cycloaddition of a SCy5-alkyne to the azide-containing peptide. Shortly, the SCy5-alkyne was dissolved in 100 mM phosphate buffer pH 7 and a 2-fold excess of the peptide was added. Premixed CuSO_4_ (12.5 µL, 20 mM) and THPTA (25 µL, 50 mM) was added, followed by aminoguanidine hydrochloride (50 µL, 100 mM). The reaction was started by the addition of sodium ascorbate (50 µL, 100 mM) and carried out overnight. Prior to purification via reverse-phase HPLC, 5 mg of DTT were added to the reaction vessel as reducing agent.

CPP conjugation

Live probe (1.0 eq.) and CPP (1.2 eq.) were dissolved in DMSO to a final concentration of 1 mM. The reaction mixture was incubated at 30 °C for 48 h and diluted with water containing 0.1% FA before purification via reverse-phase HPLC as described below.

Purification and characterization of peptides

The crude peptides and peptidic compounds were purified by reverse phase (RP) HPLC using a water-acetonitrile gradient with 0.1% formic acid (FA). LC-MS validation was performed with a similar gradient and LC-MS grade solvents. Semi-preparative HPLC was performed on a Shimadzu Prominence instrument (Shimadzu Deutschland GmbH, Duisburg, Germany) equipped with a diode-array detector (DAD) system using a C18 RP column (Onyx Monolithic HD-C18 100×4.6 mm or Onyx Monolithic C18 100×10 mm; Phenomenex, Aschaffenburg, Germany). Purity and structural identity of the peptides were verified using a DAD equipped 1260 Infinity II HPLC device with a C18 RP column (Onyx Monolithic C18 50×2 mm), coupled to a mass selective detector with a single quadruple system (Agilent Technologies, Santa Clara, CA, US) in ESI+ mode.

HEK293 Cell Cultures and Transfection

HEK293 cells were cultured in DMEM (GIBCO), supplemented with GlutaMax and pyruvate (GIBCO), 10% fetal bovine serum (GIBCO) and 1% penicillin/streptomycin (Sigma) at 37°C under 95% O_2_ / 5% CO_2_. Stable HEK293 cells expressing gephyrin-eGFP were grown with 0.4 mg/mL of the selective antibiotic G418. The cells were plated on 0.15 mm thick 18 mm glass cover slips coated with 35 µg/ml poly-D-lysine in a 12-well plate and stained and imaged the following day.

Live cell labelling of HEK293 cells stably expressing gephyrin-eGFP

For the preincubation protocol of iSylive (1) – (3), 1 eq. of the probe was mixed with 5 eq. of TNB-CPP in PBS and incubated at RT for 15 min. After the incubation, the solution was diluted 1:4 with DMEM. The HEK293 cells were washed thrice with warm PBS and incubated either at 37 °C or 4 °C with the premixed staining solution for 30 min. Before imaging, the cells were washed thrice with PBS and imaged in phenol red free DMEM at RT. For live cell labelling of the isolated iSylive-CPP, the HEK293 cells were washed thrice with warm PBS and directly incubated either at 37 °C or 4 °C with 5 µM iSylive-CPP and 25 µM TNB-CPP for 30 min, if not stated otherwise. Before imaging, the cells were washed thrice with PBS and imaged in phenol red free DMEM at RT.

Wide Field Fluorescence Microscopy of HEK293 cells stably expressing gephyrin-eGFP

The cover slips with the cell samples were inserted in an imaging chamber (Ludin Chamber Type 1, Life Imaging Services) and imaged in phenol red free DMEM. The measurements were taken from distinct samples with a sample size ≥ 2, for each group. A series of images, used to generate the data points, were acquired from different regions of the sample, each region having a distinct group of cells. The samples were imaged on an inverted Leica DMI6000B microscope with a 100x oil-immersion objective (NA 1.49) using a Leica DFC9000 GTC VSC-05760 sCMOS camera (16-bit, 2x2 binning, image pixel size: 130 nm). The 628/40 excitation and 692/40 emission filter were used for iSylive, 10 images were acquired at a frame rate (exposure time) of 100 ms and constant illumination intensity to ensure comparability.

Dissociated Hippocampal Neuronal Cultures

For iSylive staining of endogenous gephyrin, primary hippocampal neurons were isolated from embryonic brain of wild-type C57BL6 mice on day 16 (E16). Cells were seeded onto 12-mm glass coverslips coated with poly-L-lysine (5 µg/ml in PBS) and then cultured in vitro (DIV) for 14–18 days at 37°C in a humidified incubator with 5% CO2 using growth medium (Lonza, Cat# CC-4461).

Primary neurons were isolated from brain tissue of E18 C57Bl/6J mice, trypsin digested (0.05%, ThermoFisher 25300054) and triturated with pipette tips of differing pore sizes. Isolated neurons were plated on 24 mm high precision coverslips coated with 600 µl Poly-D lysine (0.1 mg/ml, Sigma Aldrich) at 4 °C over weekend. Neurons were supplemented with 1.6 ml of Neurobasal medium containing 1% Glutamax, 2% B27 Plus supplement (Life Technologies), and 5 μg/ml gentamycin (Sigma Aldrich). 50 % of culture medium was replaced weekly.

For live iSylive labelling of mEos4b-gephyrin in lentivirus infected neurons, we used dissociated hippocampal neurons isolated from E17.5 embryonic mice. After trypsinisation and trituration with 0.3 mg/ml DNase I (Merck, #11284932001) in plating medium composed of Minimal Essential Medium (MEM) containing Earle’s Balanced Salts (EBSS) (Cytiva, #SH30244.01), 2 mM GlutaMAX (Gibco, #35050-038), 1 mM sodium pyruvate (Thermo Fisher Scientific, #11360-039), and 10% heat inactivated horse serum (Gibco, #26050-088), cells were seeded at 10^5^ cells/well in 12 well plates on round glass coverslips (type 1.5, 18 mm diameter; Marienfeld, #0112580) that had been pre-coated with 80 µg/ml poly-D,L-ornithine (Merck, #P8638).The medium was replaced 3 hours after plating with neurobasal medium (Gibco, #21103-049) containing B-27 Supplement (Gibco, #17504-044) and 2 mM GlutaMAX. Once a week, 300 µL of fresh maintenance medium was added per well. Neurons were infected at DIV4 with lentivirus preparation (5 µl) for the expression of full-length rat gephyrin (splice variant P1, GenBank X66366) tagged at its N-terminus with mEos4b (construct FU-mEos4b-gephyrin^1^).

For eSylive staining, dissociated hippocampal neurons of the early postnatal (P0–P1) wild-type C57black/6J mice were prepared as previously described^2^ and cultured in a Neurobasal medium (cat no. 21103049, Thermo Fisher Scientific) supplemented with 1× N21-Max (cat no. AR008, R&D systems), 1× GlutaMax Supplement (cat no. 35050061, Thermo Fisher Scientific), 5% heat inactivated fetal bovine serum (cat no. 35-010-CV, Corning), and 1 μg/mL gentamycin (cat no. G1272, Sigma-Aldrich). Neurons were plated on poly-l-lysine (cat no. 3056, Peptide Institute)-coated coverslips (25 mm, #1.5; cat no. 64-0715, Warner Instruments), pretreated with nitric acid with a density 200,000/well in 6-well plates and were maintained in a humidified 37 °C incubator with 5% CO2 until 20–22 DIV.

Immunocytochemistry

For iSylive stainings of endogenous gephyrin, primary neurons were labeled with iSylive/TNB-CPP using the indicated concentrations, incubated for 30 min at 4 °C and then fixed in 4% paraformaldehyde with 4% sucrose. After fixation, coverslips were washed with PBS and processed for immunocytochemical staining. Cells were permeabilized and blocked with 10% normal goat serum in PBS + 0.25% Triton X-100 before being incubated overnight at 4°C with primary antibodies (Gephyrin 3B11, Synaptic Systems #147111, RRID: AB_2619837, Dilution 1:500). The cells were then washed three times in PBS and incubated with secondary antibodies for 1 hour.

iSylive labelling on mEos4b-gephyrin infected neurons was carried out on DIV15. iSylive and TNB-CPP were resuspended in PBS with 1% DMSO at a concentration of 0.5 mM and 5 mM, respectively, mixed and diluted 40× in PBS and incubated for 15 min at 30°C. The iSylive/TNB-CPP reaction mix was diluted 25× in Tyrode solution (120 mM NaCl, 2.5 mM KCl, 2 mM CaCl_2_, 2 mM MgCl_2_, 25 mM glucose, 5 mM pyruvate, 25 mM HEPES, pH 7.4), applied to the cells at a final concentration of 0.5 µM iSylive and 5 µM TNB-CPP, and incubated for 30 min at room temperature. After three washes, the cells were imaged in Tyrode solution.

Epifluorescence microscopy was done as described previously^3^ with a Zeiss ELYRA PS.1 microscope using a Plan-Apochromat 100x oil-immersion objective (N.A. 1.46), an 1.6× lens in the emission path, and an Andor iXon 897 EMCCD camera with a final image pixel size of 100 nm. mEos4b-gephyrin was detected using a 488 nm laser at 0.2% nominal power, an exposure time of 100 ms, and a camera gain of 300. iSylive was imaged with 10% of a 642 nm laser, 100 ms exposure time 200 gain. Three image frames were recorded and averaged for each field of view. Seven infected cells expressing mEos4b-gephyrin were analysed. The correlation between mEos4b-gephyrin and iSylive puncta was calculated using the JACoP plugin in ImageJ.

For eSylive staining, for 20–22 DIV mouse hippocampal neurons cultured in μ-slides (ibidi, Cat. #80807), half of the culture medium was removed and kept at 37 °C for later use. A 1000× stock solution containing 100 µM eSylive and 2 mM TNB-CPP was freshly prepared in ultrapure water. The eSylive/TNB-CPP premix was added to the culture medium at a 1:1000 dilution, and the cells were incubated for 30 min at 37 °C in a CO₂ incubator. After incubation, the eSylive-containing medium was removed and replaced with the previously saved culture medium, and the cells were allowed to recover for 30 min at 37 °C. After recovery, the μ-slides were rinsed twice with PBS and imaged directly within 10 min. The samples were subsequently fixed with 3% glyoxal solution for 20 min as previously described^4^, followed by three washes with PBS. Blocking of nonspecific binding and permeabilization were performed by incubating the neurons in blocking solution containing 50% Fish Serum Blocking Buffer (Thermo Fisher Scientific, Cat. #37527) and 0.5% Triton X-100 in PBS for 1 h. Neurons were then incubated with PSD-95 antibody (K28/43, 1:500) diluted in blocking buffer for 2 h at room temperature. After three washes with PBS, the samples were incubated with Alexa Fluor 488–conjugated secondary antibody (Thermo Fisher Scientific, Cat. #A11001) for 1 h. Finally, the cells were washed three times with PBS and imaged directly.

Confocal microscopy for fixed neuronal culture

Coverslips were embedded in Aqua Poly Mount and analyzed using a Nikon microscope equipped with a spinning disk (Yokogawa, Visitron Systems), solid-state lasers (488, 647), objectives (100×, 60x), and two EM-CCD cameras (Hamamatsu Photonics 512/1024×).

Multi-color confocal imaging of eSylive was performed on a Leica TCS SP8 gated STED 3X microscope equipped with a 500 mW tunable white-light laser (470–670 nm). Images were acquired using gated HyD detectors (gain = 100) and a 63× oil-immersion objective (Leica HC PL APO 63×/1.40 Oil CS2). A 0.3 ns time gate was applied to reduce stray scattered light. Z-stacks were collected with a voxel size of 0.0946 × 0.0946 × 0.2985 µm³.

Image processing was performed in ImageJ/Fiji. Z-stacks were first denoised using a 3D median filter (radius = 1 × 1 × 2 in x/y/z), followed by 3 × 3 binning in the xy plane to generate near-isotropic voxel sampling. Fourier analysis (FFT) was used to confirm that binning did not reduce optical resolution. To enhance synaptic structures, a 3 × 3 median-filtered duplicate image was subtracted from the binned image.

Colocalization analysis was performed on the 3D stacks using the ImageJ Colocalization Test, with significance assessed by randomization using the Fay method (x, y, z translations) with 75 iterations.

2D image processing and analysis

Image processing and analysis were carried out using Fiji (**F**iji **I**s **J**ust **I**mageJ)^5^ with JACoP (**J**ust **A**nother **Co**localization **P**lugin)^6^ plugin for colocalization analysis. For HEK293 cell image analyses, macros and scripts were written by V.K.

**Supplementary Figures**

Supplementary Figure **1****
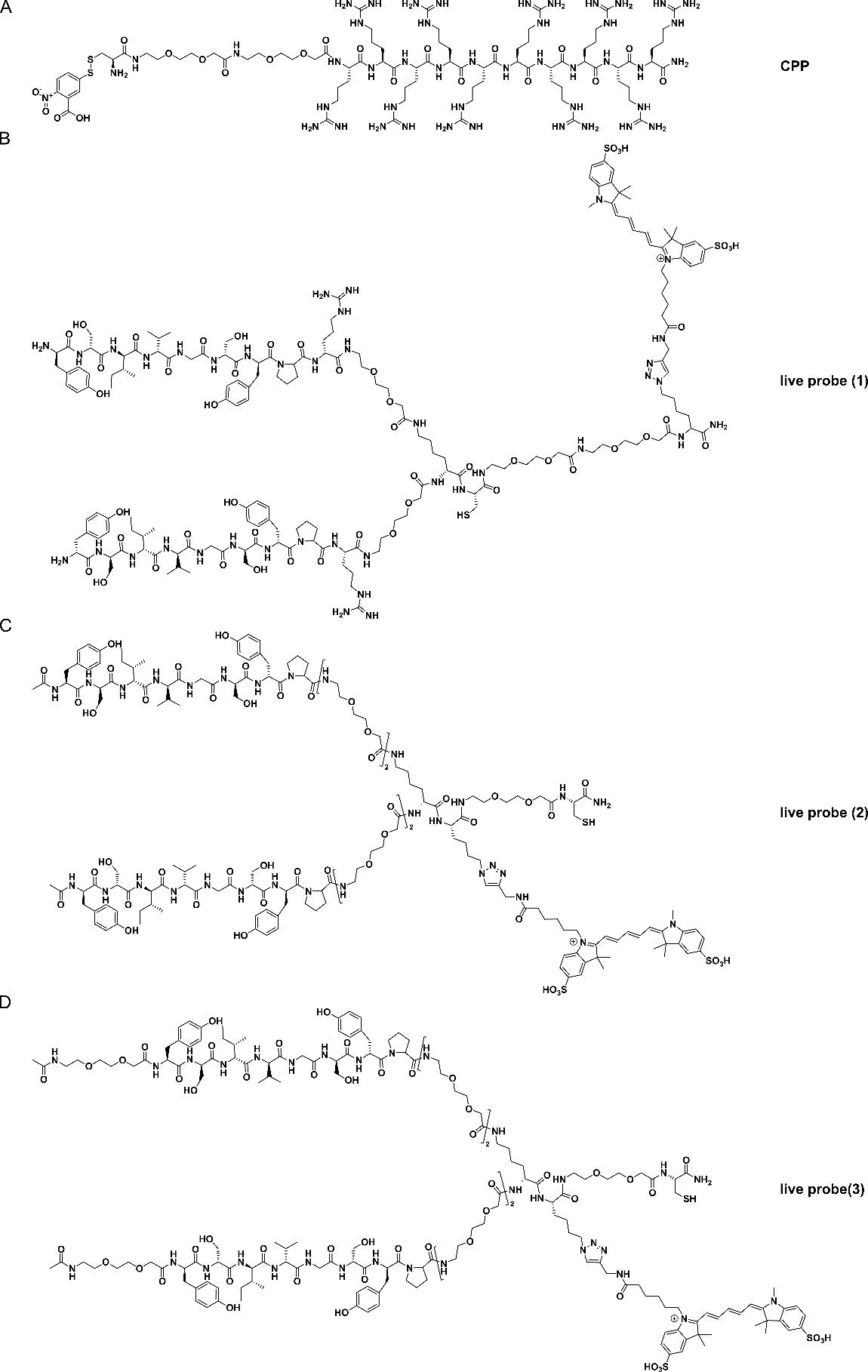
**

**Figure S1. Chemical structures of the iSylive (1-3) probes and TNB-CPP corresponding to Figure 2.** Shown are the chemical structures of TNB-CPP (A), live probe (**1**) (B), live probe (**2**) (C) and live probe (**3**) (D) that are schematically introduced in Figure 2.

Supplementary Figure **2**


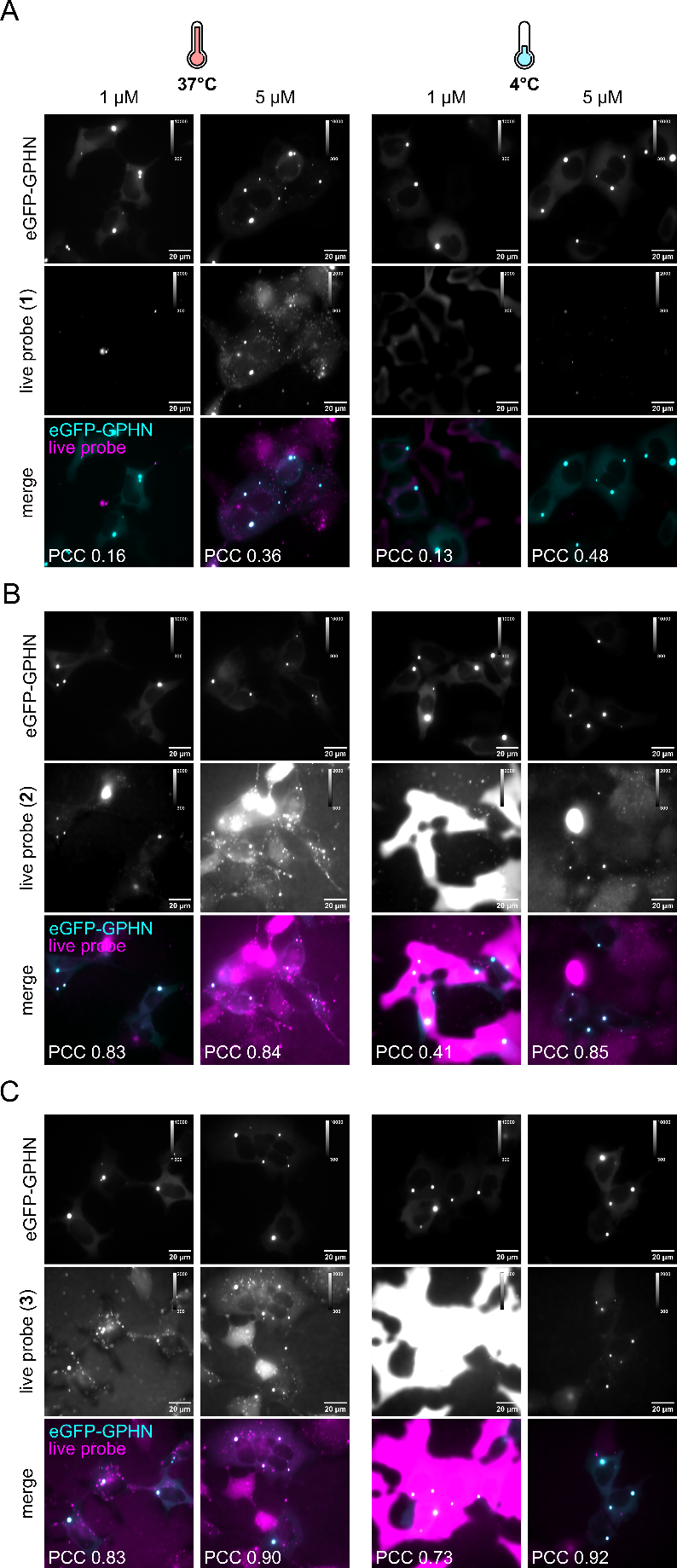


**Figure S2. Microscopic evaluation of live probes (1) – (3) corresponding to Figure 2.** Representative images of (A) live probe (1), (B) live probe (2) and (C) live probe (3) staining of HEK293 cells stably expressing eGFP-gephyrin at 4 °C or 37 °C following 15 min co-incubation with different concentrations of the live probe and 5 eq. of the TNB-CPP, respectively. The same contrast was applied to alle images to enable comparability.

Supplementary Figure **3**


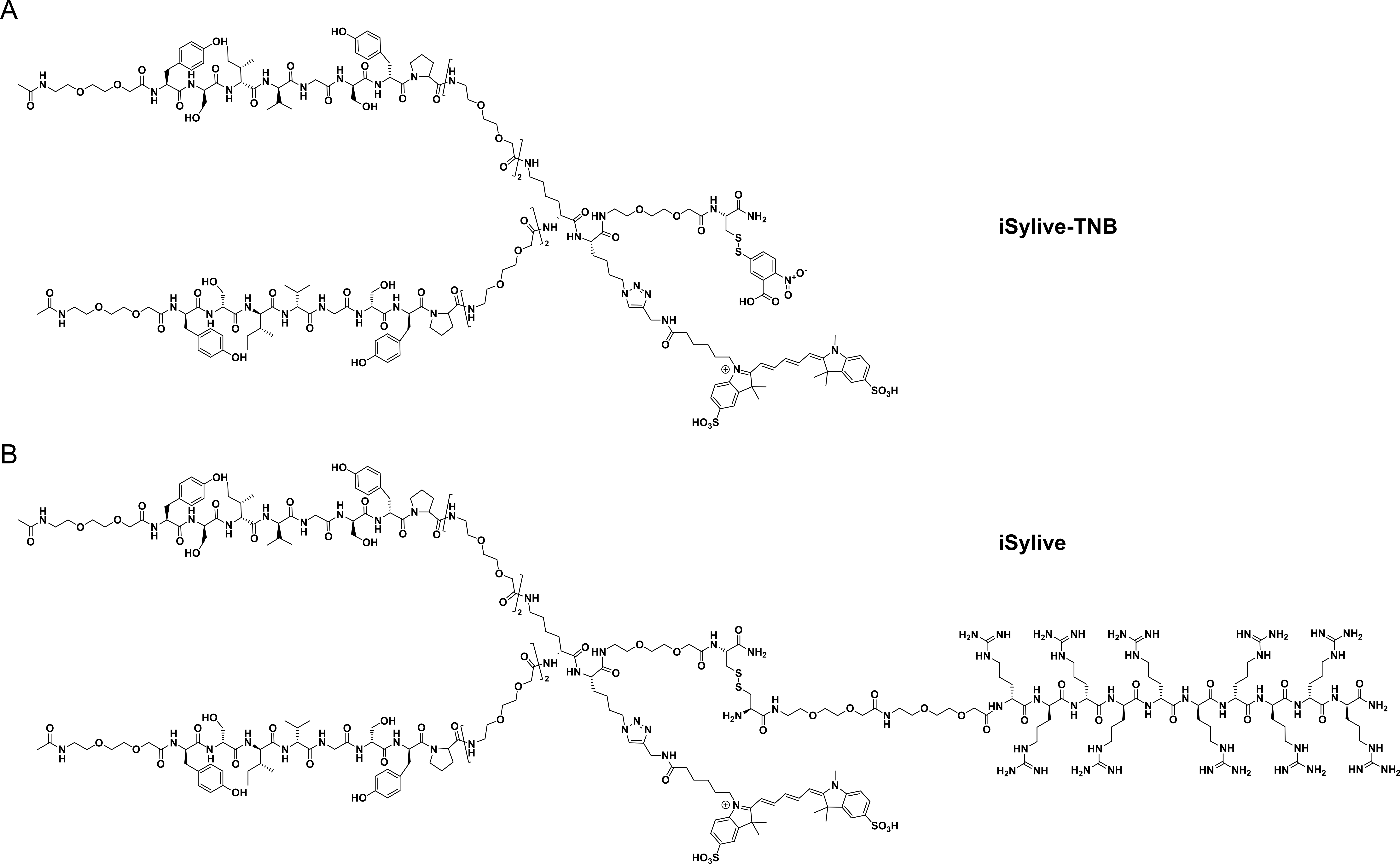


**Figure S3. Chemical structures of the iSylive probes corresponding to Figure 3.** Shown are the chemical structures of iSylive-TNB (A) and iSylive (B) that are schematically introduced in Figure 3.

Supplementary Figure **4**


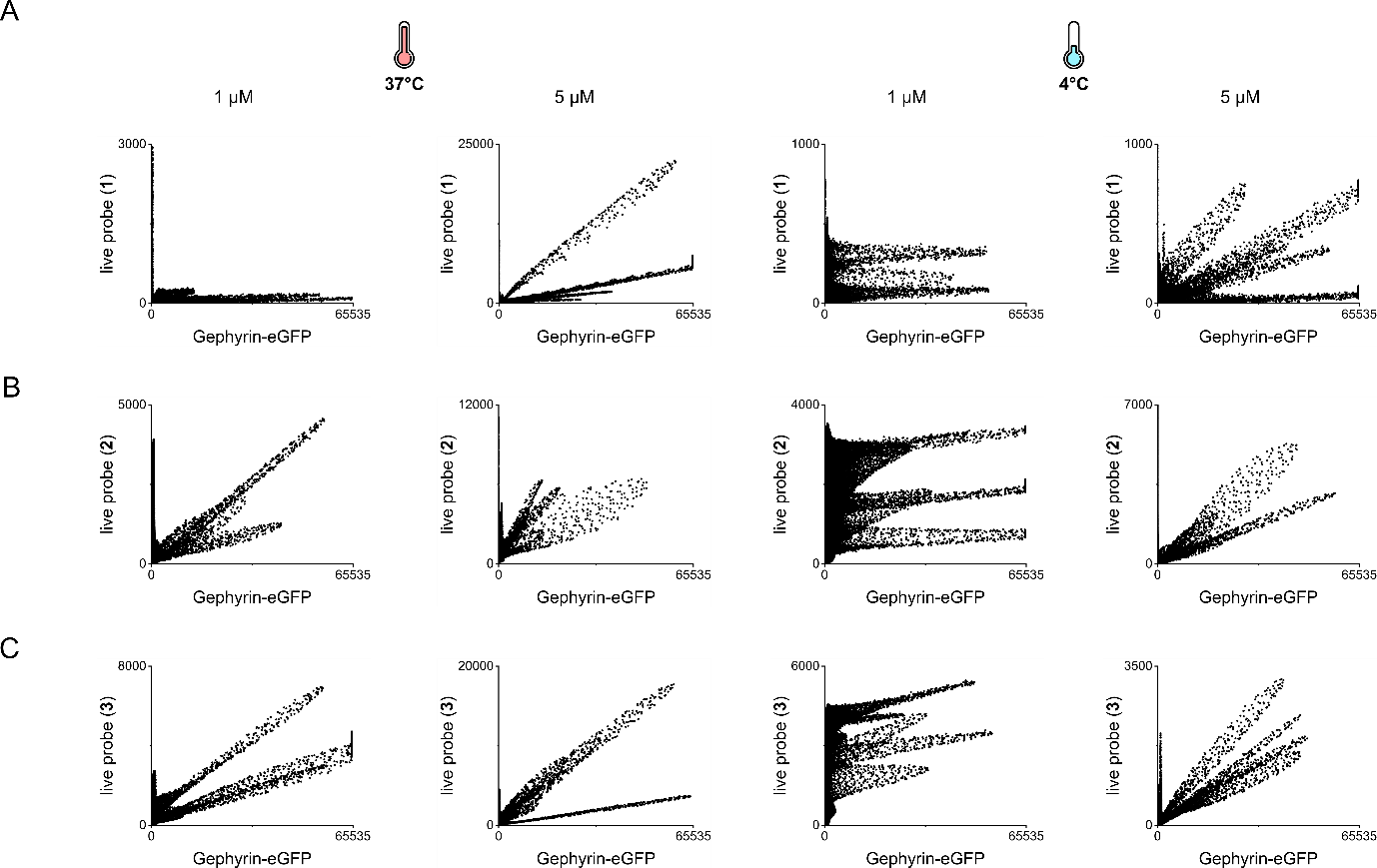


**Figure S4.** **Microscopical analysis of live probe performance in HEK293 cells corresponding to Figure 2.** Cytofluorogram showing the intensity correlation of fluorescent-tagged gephyrin versus affinity probe labelled gephyrin of (A) live probe (**1**), (B) live probe (**2**) and (C) live probe (**3**) (arbitrary units, a.u.) corresponding to the images shown in Figure 2.

Supplementary Figure **5**


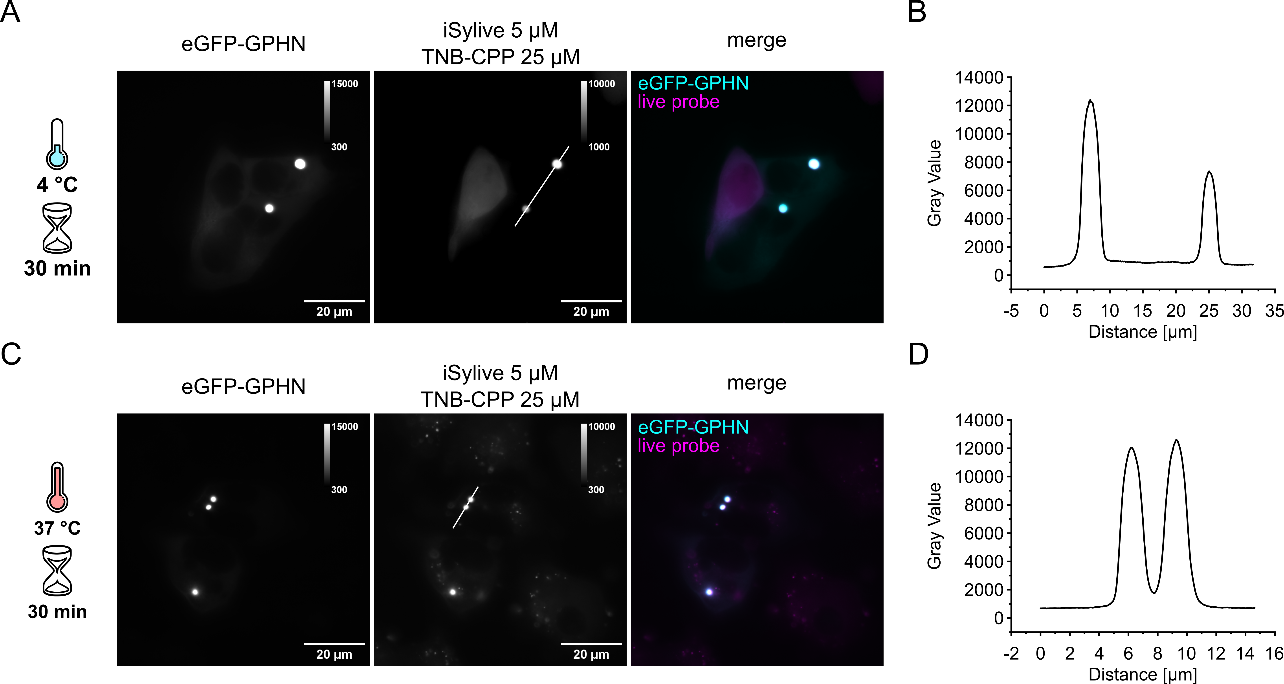


**Figure S5. Microscopic evaluation of iSylive at different incubation temperatures.** (A) Representative images of iSylive staining of living HEK293 cells expressing eGFP-gephyrin. The cells were directly stained with 5 µM iSylive and 25 µM TNB-CPP for 30 min at 4 °C. (B) Line profile along the white line in the respective image in (A). (C) Representative images of iSylive staining of living HEK293 cells expressing eGFP-gephyrin. The cells were directly stained with 5 µM iSylive and 25 µM TNB-CPP for 30 min at 37 °C. (D) Line profile along the white line in the respective image in (C).

Supplementary Figure **6**


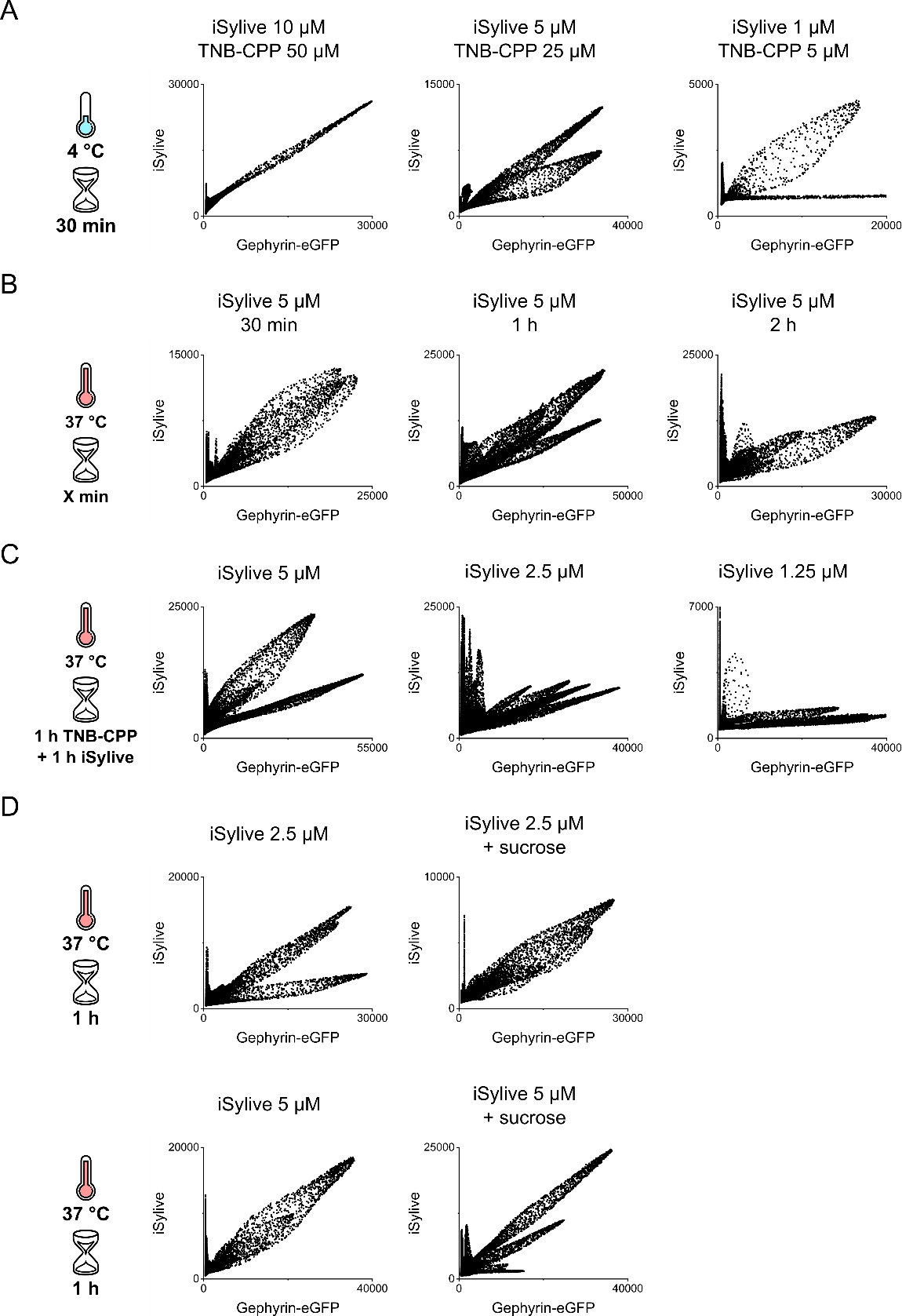


**Figure S6. Microscopical analysis of iSylive performance in HEK293 cells corresponding to Figure 4.** Cytofluorogram showing the intensity correlation of fluorescent-tagged gephyrin versus affinity probe labelled gephyrin corresponding to the images shown in Figure 4. (A) The cells were directly stained with 5 µM iSylive and 25 µM TNB-CPP for 30 min at 4 °C. (B) The cells were directly stained with varying iSylive and TNB-CPP concentrations for 30 min at 4 °C. (C) The cells were directly stained with 5 µM iSylive and 25 µM TNB-CPP for varying incubation times at 37 °C. (D) The cells were incubated for 1 h with 25 µM TNB-CPP before varying concentrations of iSylive were applied for another 1 h at 37 °C. The cells were incubated with (right) or without (left) 450 mM sucrose in addition to varying concentrations of iSylive and 25 µM TNB-CPP for 1 h at 37 °C.

Supplementary Figure **7**


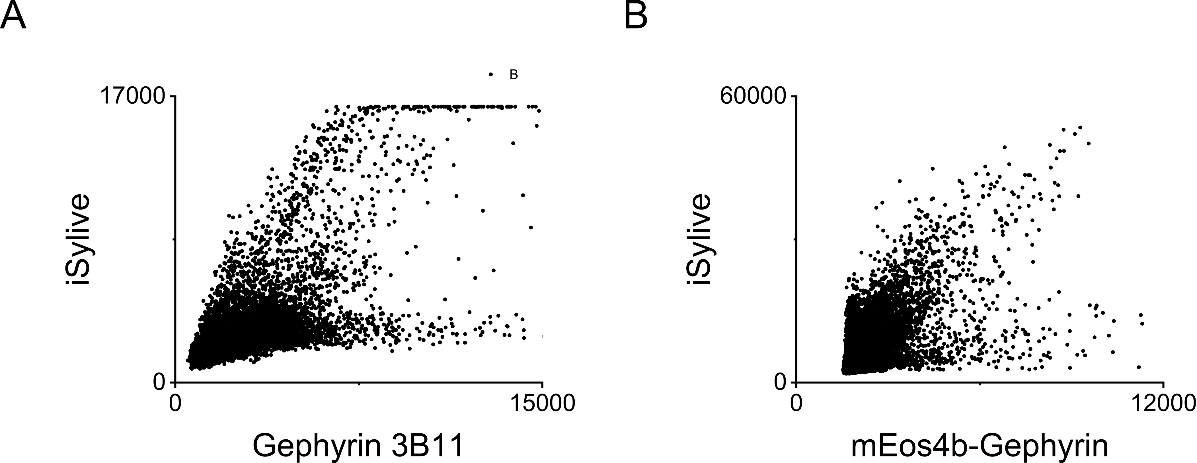


**Figure S7. Microscopical analysis of iSylive performance in hippocampal neurons corresponding to Figure 5.** (A) Cytofluorogram showing the intensity correlation of immunolabelled gephyrin versus iSylive labelled gephyrin in primary neurons corresponding to the images shown in Figure 5C. (B) Cytofluorogram showing the intensity correlation of mEos4b tagged gephyrin versus iSylive labelled gephyrin in lentivirus infected neurons corresponding to the images shown in Figure 5F.

Supplementary Figure **8**


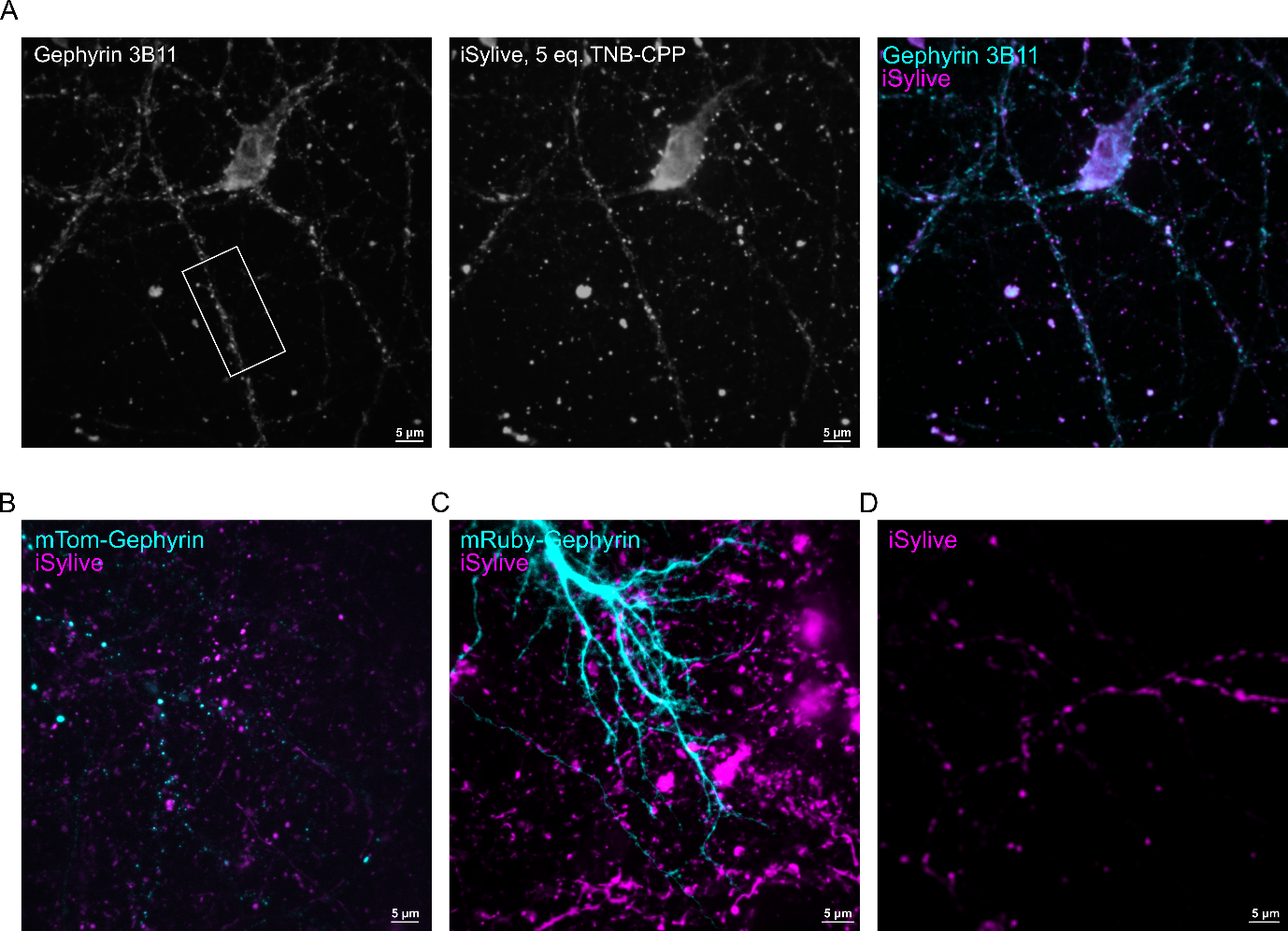


**Figure S8. iSylive application in living mouse primary transfected and untransfected neurons.** (A) iSylive labelling of endogenous living neurons followed by fixation and antibody co-staining. Full size images of the ROIs shown in Figure 2B. (B) iSylive labelling of mTOM-Gephyrin transfected neurons showing aggregation of the probe. (C) iSylive labelling of mRuby transfected neurons showing aggregation of the probe. (D) iSylive labelling of endogenous living neurons showing a punctate distribution.Supplementary Figure **9**


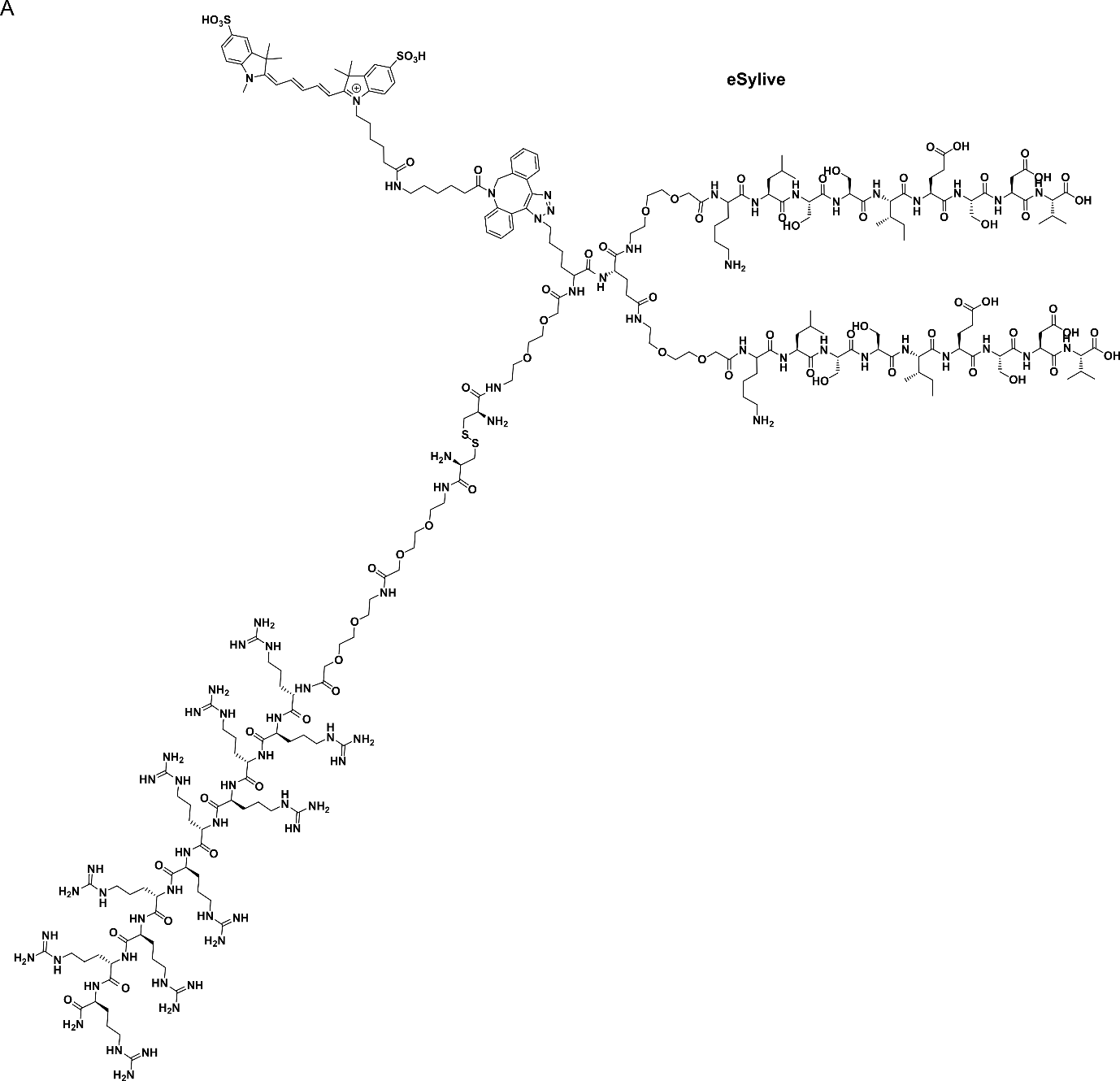


**Figure S9. Chemical structures of the eSylive probe corresponding to Figure 6.** Shown is the chemical structure of eSylive that is schematically introduced in Figure 6.

Supplementary Figure **10**


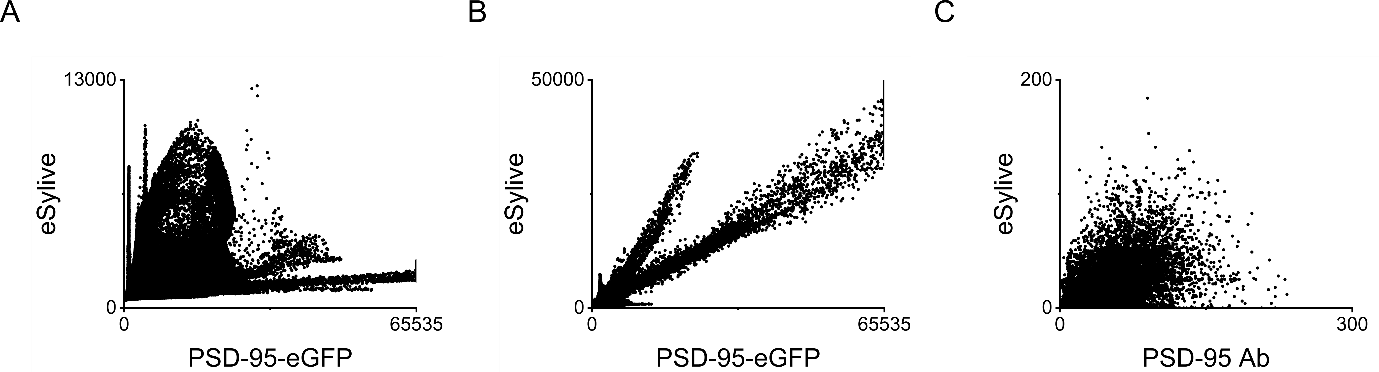


**Figure S10. Microscopical analysis of eSylive performance in HEK293 cells and primary hippocampal neurons corresponding to Figure 6.** (A) Cytofluorogram showing the intensity correlation of eGFP tagged PSD-95 versus eSylive labelled PSD-95 in HEK293 cells corresponding to the images shown in Figure 6B (37 °C). (B) Cytofluorogram showing the intensity correlation of eGFP tagged PSD-95 versus eSylive labelled PSD-95 in HEK293 cells corresponding to the images shown in Figure 6B (4 °C). (C) Cytofluorogram showing the intensity correlation of immunolabelled PSD-95 versus eSylive labelled PSD-95 in primary neurons corresponding to the images shown in Figure 6C.

Supplementary Figure **11**


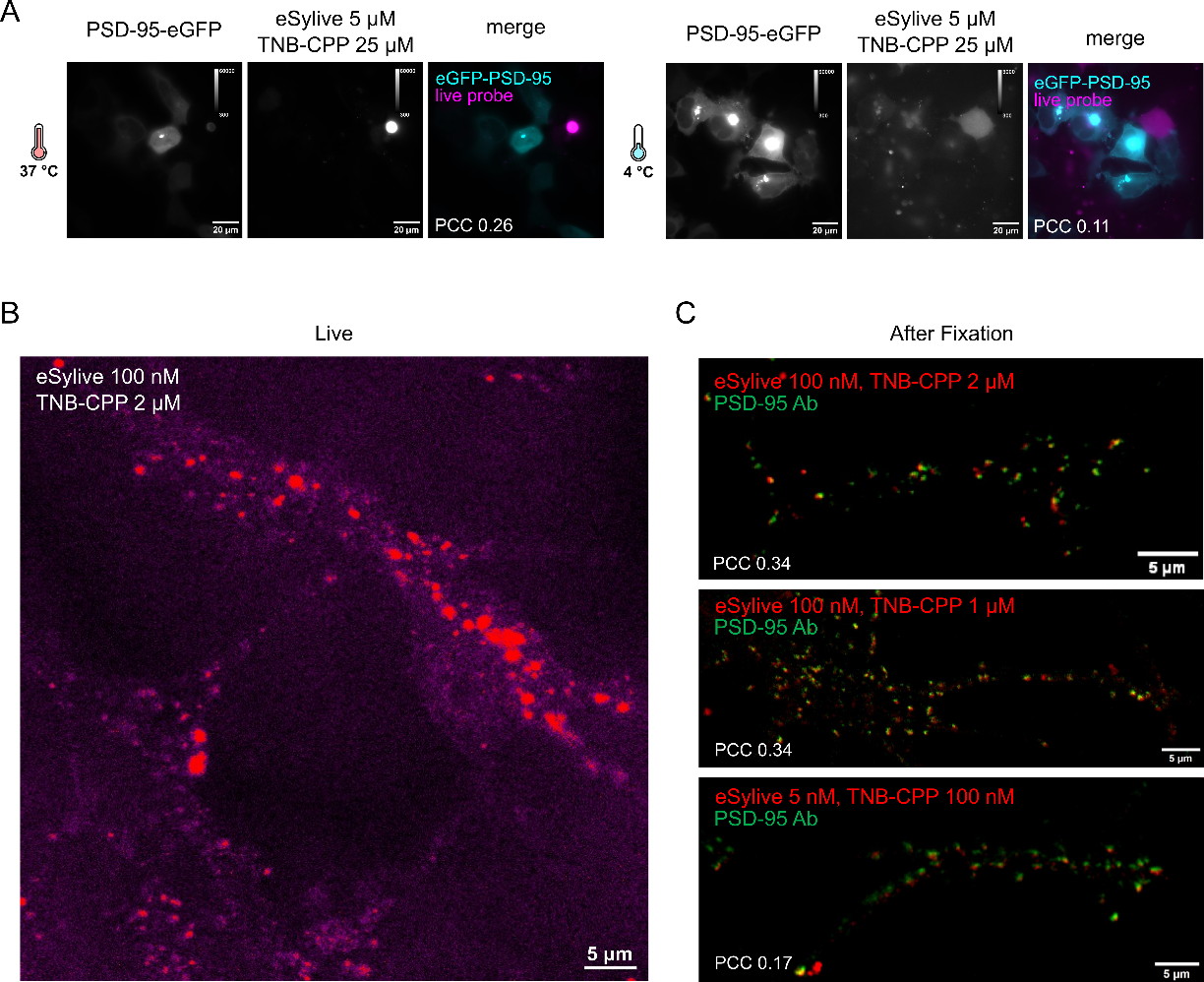


**Figure S11. eSylive application in living mouse primary untransfected neurons.** Representative images of eSylive staining of living HEK293 cells expressing PSD-95-eGFP. The cells were directly stained with 5 µM eSylive and 25 µM TNB-CPP for 30 min at either 37 °C (left) or 4 °C (right). (B) eSylive labelling of endogenous living neurons. (C) eSylive labelling of endogenous living neurons using different probe concentrations followed by fixation and antibody co-staining showing a punctate distribution and high degree of colocalization.

**Supplementary Tables**

Supplementary Table S1. Mass spectrometric probe validation.

| Compound | Sequence | Purity (%) | Chemical formula | Calculated m/z | Observed m/z |
| --- | --- | --- | --- | --- | --- |
|  |  |  |  |  |  |
| live probe (**1**) | (YSIVGSYPR-O2Oc)_2_-KC-O2Oc-O2Oc-K(N_3_)-NH_2_ | >95% | C_170_H_258_N_39_O_48_S_3_^+^ | 928.5 [M+3H]^4+^ | 928.1  [M+3H]^4+^ |
| live probe (**2**) | Ac-(YSIVGSYP-O2Oc-O2Oc)_2_-K-K(N_3_)-O2Oc-C-NH_2_ | >95% | C_168_H_248_N_32_O_51_S_3_^+^ | 1209.2 [M+2H]^3+^ | 1209.8  [M+H]^2+^ |
| live probe (**3**) | Ac-O2Oc-(YSIVGSYP-O2Oc-O2Oc)_2_-K-K(N_3_)-O2Oc-C-NH_2_ | >95% | C_180_H_269_N_34_O_57_S_3_^+^ | 1305.9  [M+2H]^3+^ | 1306.3  [M+2H]^3+^ |
| iSylive | Ac-O2Oc-(YSIVGSYP-O2Oc-O2Oc)_2_-K-K(N_3_)-O2Oc-C-NH_2_-C-O2Oc-O2Oc-(R)_10_-NH_2_ | >95% | C_255_H_417_N_78_O_74_S_4_^+^ | 841.9 [M+6H]^7+^ | 841.9 [M+6H]^7+^ |
| iSylive-TNB | Ac-O2Oc-(YSIVGSYP-O2Oc-O2Oc)_2_-K-K(N_3_)-O2Oc-C-NH_2_-TNB | >95% | C_187_H_272_N_35_O_61_S_4_^+^ | 1371.6 [M+2H]^3+^ | 1372.2 [M+2H]^3+^ |
| eSylive | C-O2Oc-K(N_3_)-E(O2Oc-KLSSIESDV)_2_-OH | >95% | C_242_H_404_N_77_O_72_S_4_^+^ | 811.0  [M+6H]^7+^ | 811.1  [M+2H]^3+^ |
| TNB-CPP | TNB-C-O2Oc-O2Oc-(R)_10_-NH_2_ | >95% | C_82_H_153_N_45_O_21_S_2_ | 434.6  [M+5H]^5+^ | 434.7  [M+2H]^3+^ |

**Supplementary References**

1. Niwa, F., Patrizio, A., Triller, A. & Specht, C.G. cAMP-EPAC-Dependent Regulation of Gephyrin Phosphorylation and GABA<sub>A</sub>R Trapping at Inhibitory Synapses. *iScience* **22**, 453-465 (2019).

2. Chen, Y. et al. NS21: Re-defined and modified supplement B27 for neuronal cultures. *Journal of Neuroscience Methods* **171**, 239-247 (2008).

3. Camuso, S. et al. Single molecule counting detects low-copy glycine receptors in hippocampal and striatal synapses. *bioRxiv*, 2025.01.07.631736 (2025).

4. Richter, K.N. et al. Glyoxal as an alternative fixative to formaldehyde in immunostaining and super‐resolution microscopy. *The EMBO Journal* **37**, 139-159 (2018).

5. Schindelin, J. et al. Fiji: an open-source platform for biological-image analysis. *Nature Methods* **9**, 676-682 (2012).

6. Bolte, S. & Cordelières, F.P. A guided tour into subcellular colocalization analysis in light microscopy. *J Microsc* **224**, 213-32 (2006).

**Appendix 1: Chromatographic and mass spectrometric probe validation**

Live probe (1)





Live probe (2)


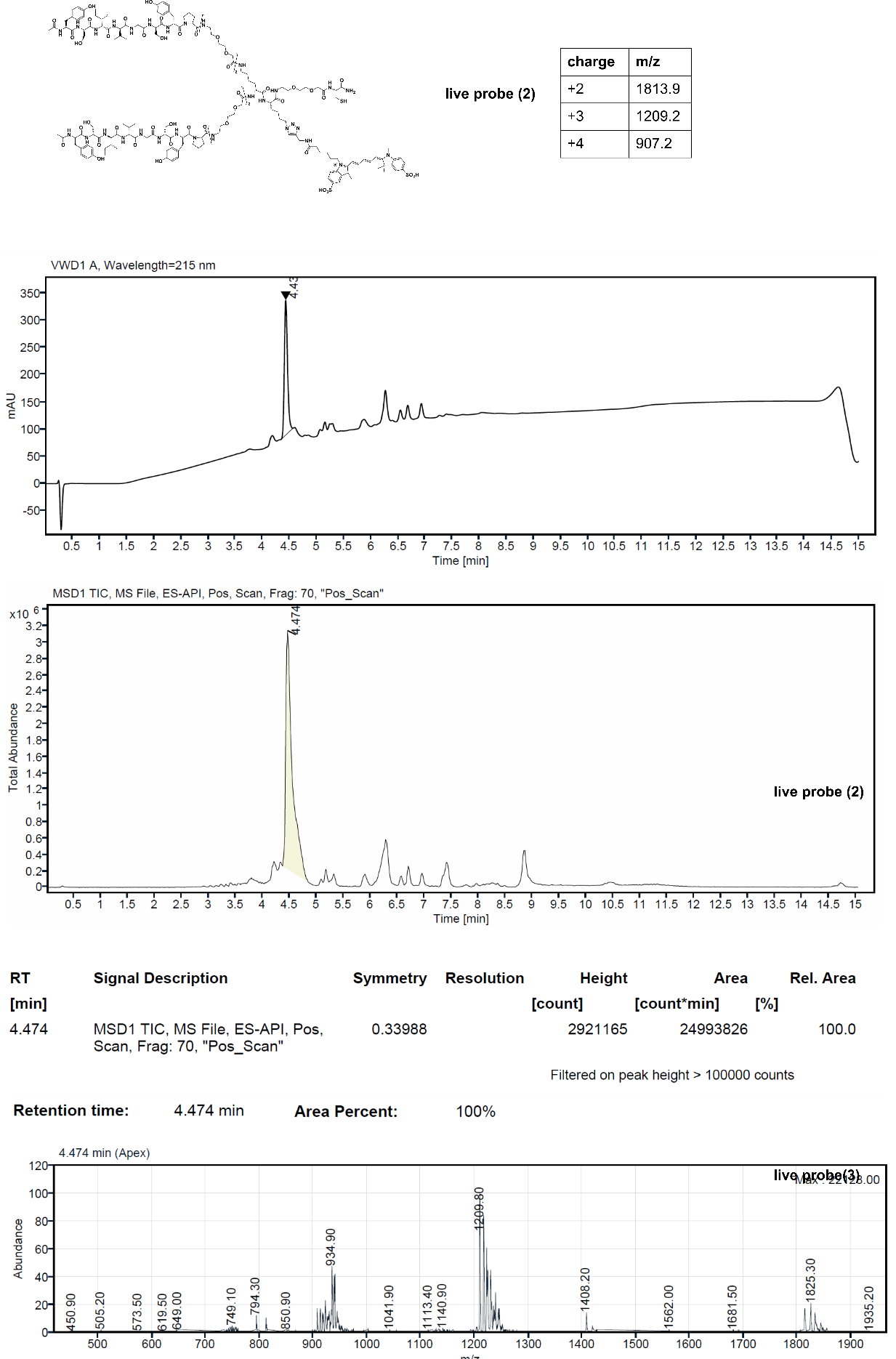


Live probe (3)


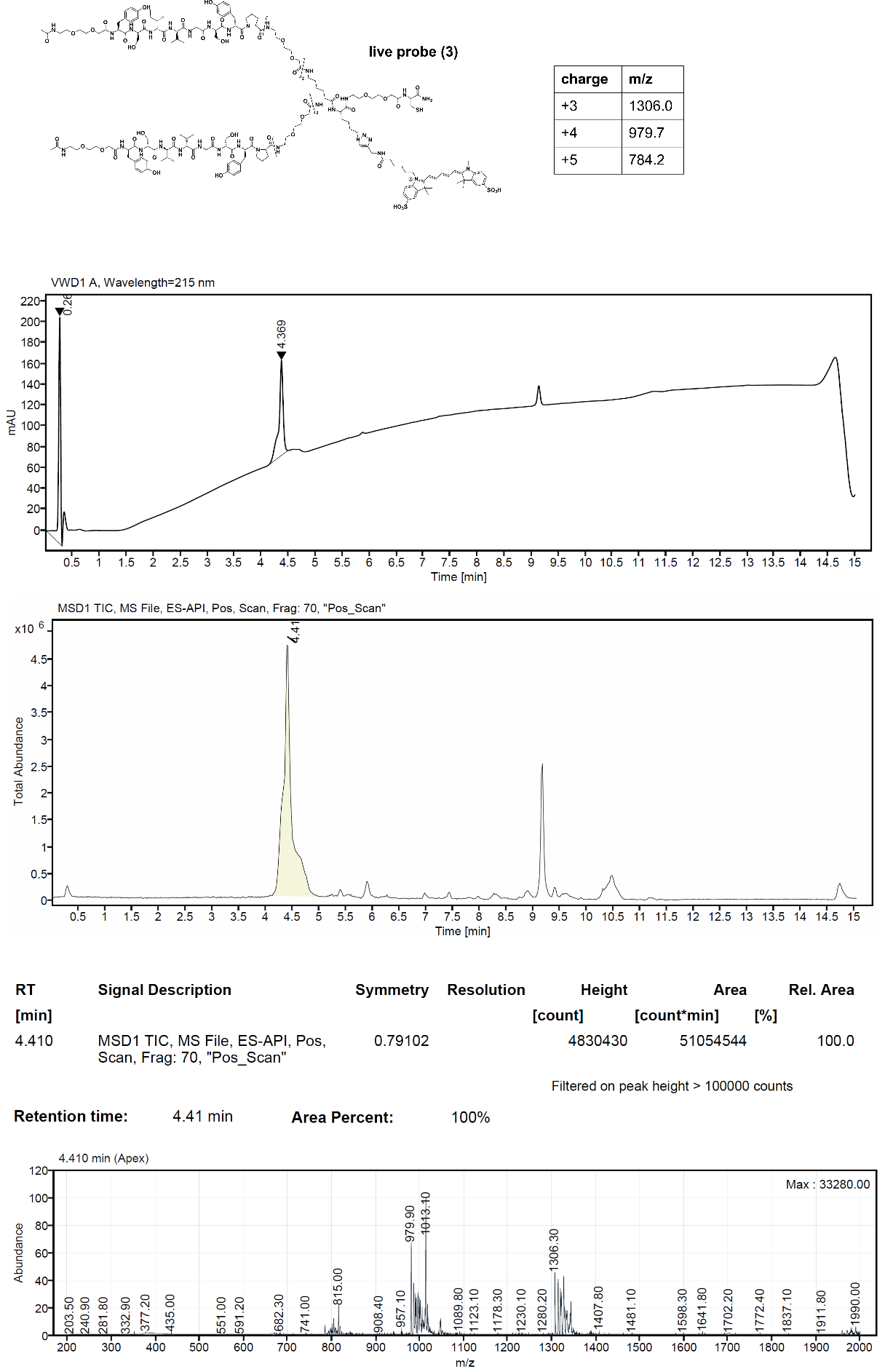


iSylive


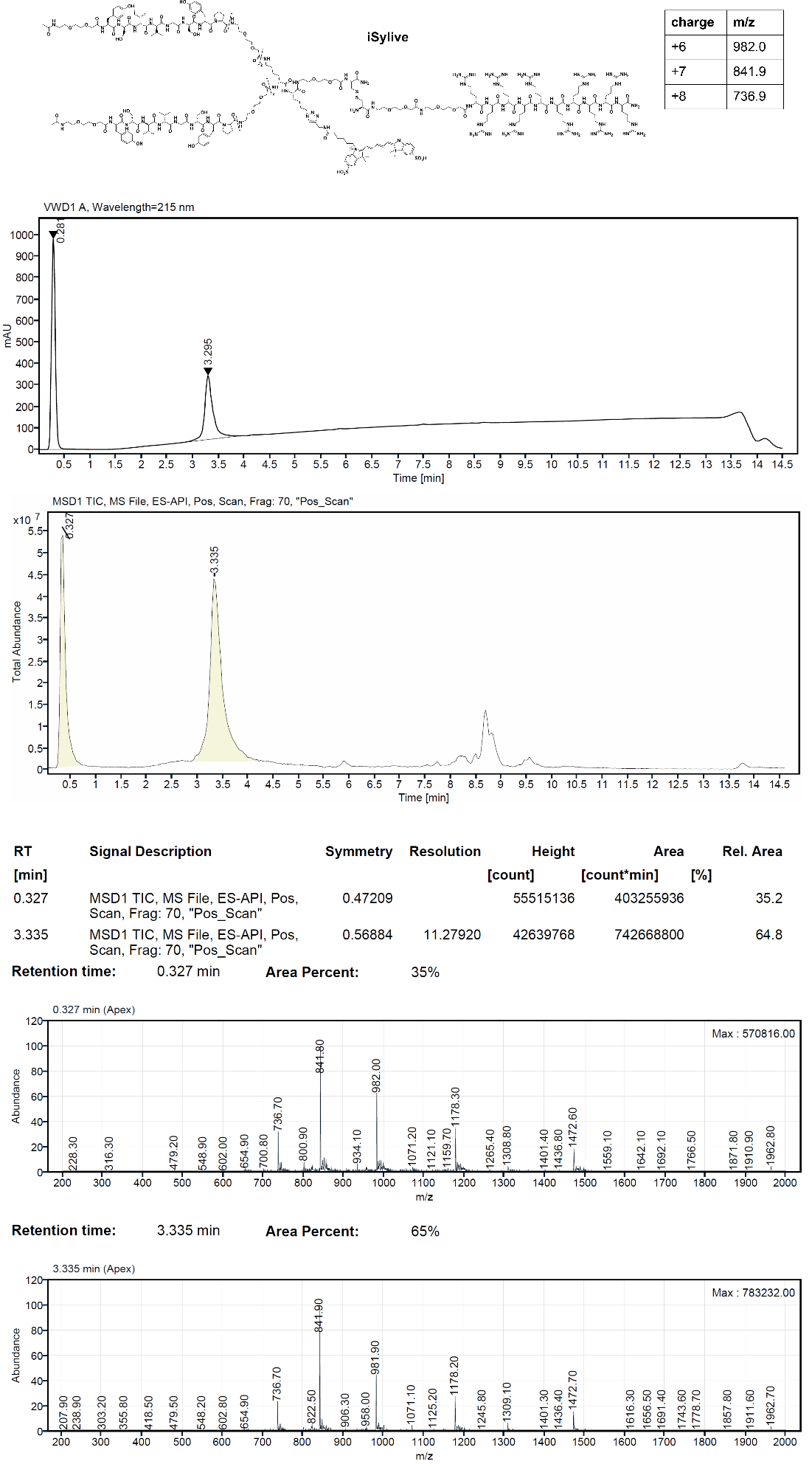


eSylive


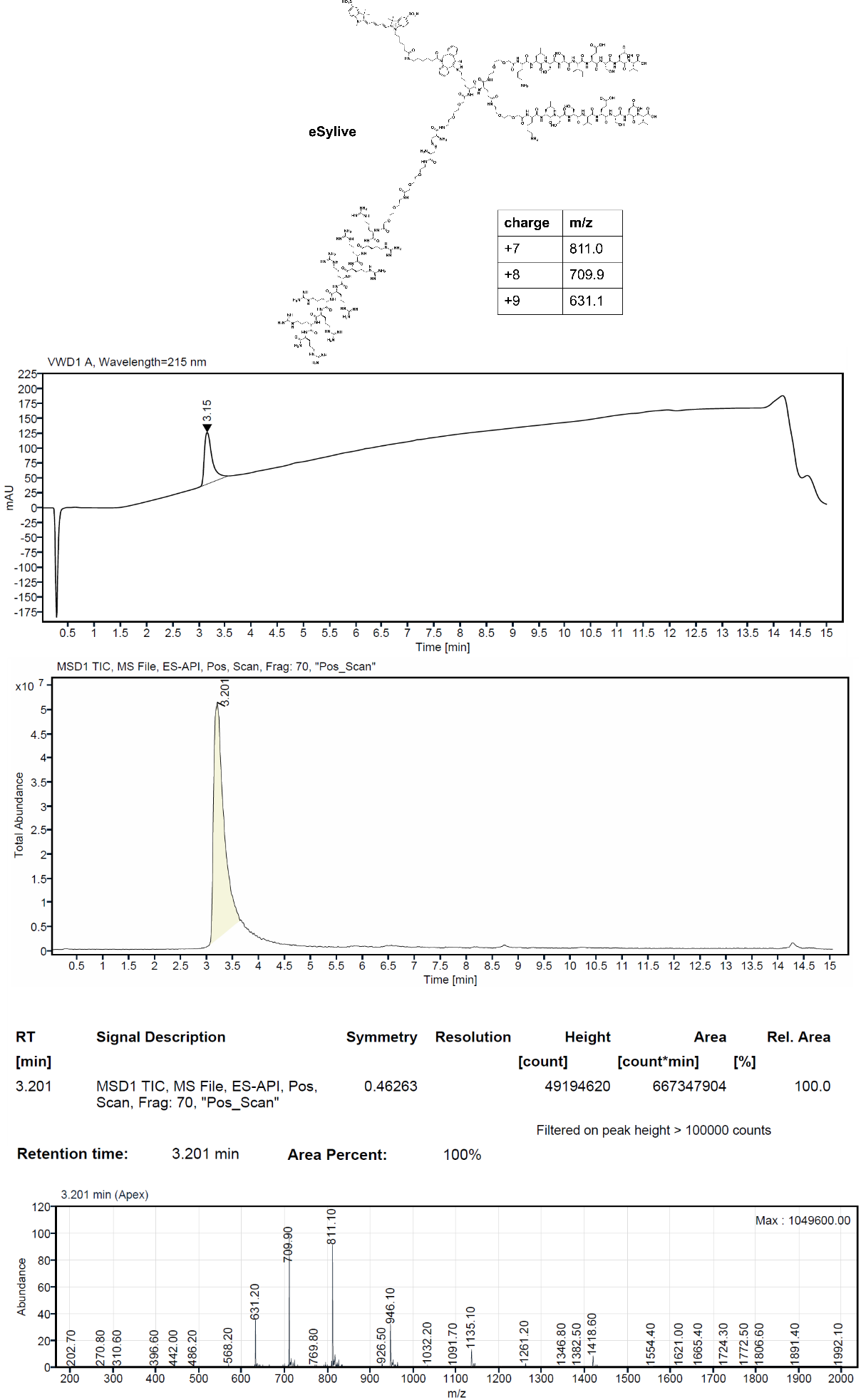


TNB-CPP





Original chromatogram and mass spectrometric validation corresponding to Main Figure 3F


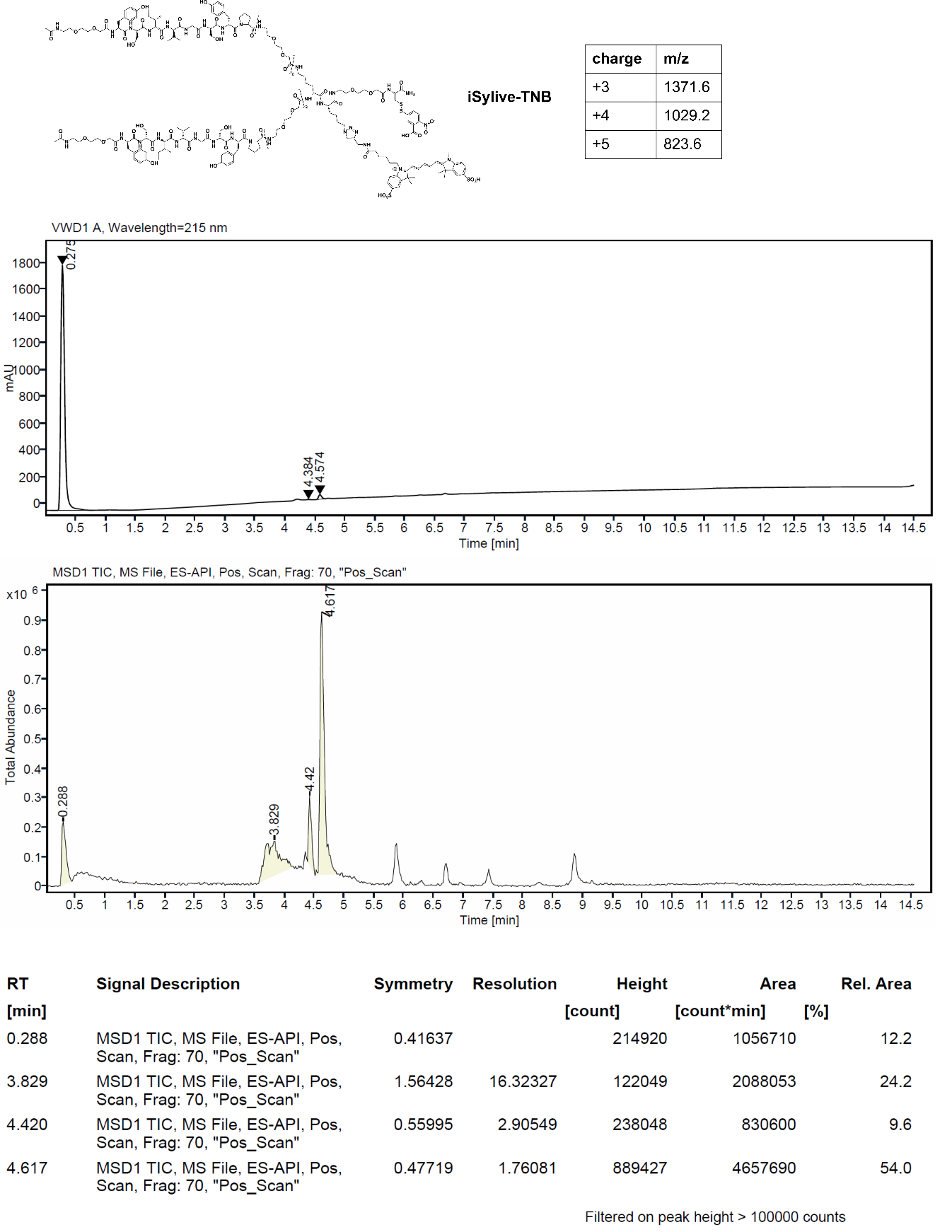


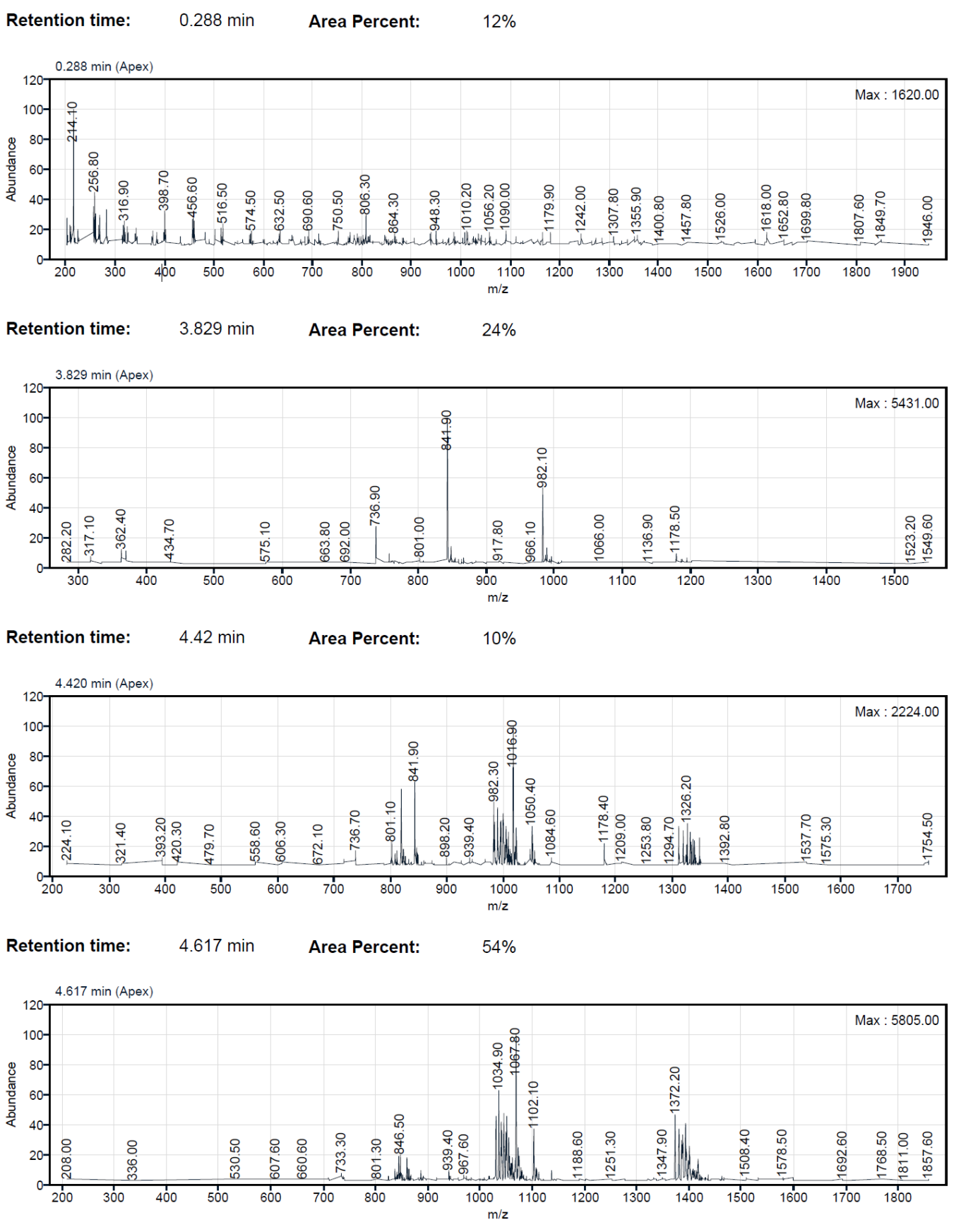

Supplement: Supplementary file 1 — Supporting File 1: anie71274‐sup‐0001‐SuppMat.docx. [file ANIE-65-e19933-s001.docx]
